# Cleavage modification did not alter blastomere fates during bryozoan evolution

**DOI:** 10.1186/s12915-017-0371-9

**Published:** 2017-04-28

**Authors:** Bruno C. Vellutini, José M. Martín-Durán, Andreas Hejnol

**Affiliations:** Sars International Centre for Marine Molecular Biology, University of Bergen, Thormøhlensgate 55, 5006 Bergen, Norway

**Keywords:** Bryozoa, Cyphonautes, Spiral cleavage, Cell lineage, Larva, MAPK, Gene expression, Molecular patterning

## Abstract

**Background:**

Stereotypic cleavage patterns play a crucial role in cell fate determination by precisely positioning early embryonic blastomeres. Although misplaced cell divisions can alter blastomere fates and cause embryonic defects, cleavage patterns have been modified several times during animal evolution. However, it remains unclear how evolutionary changes in cleavage impact the specification of blastomere fates. Here, we analyze the transition from spiral cleavage – a stereotypic pattern remarkably conserved in many protostomes – to a biradial cleavage pattern, which occurred during the evolution of bryozoans.

**Results:**

Using 3D-live imaging time-lapse microscopy (4D-microscopy), we characterize the cell lineage, MAPK signaling, and the expression of 16 developmental genes in the bryozoan *Membranipora membranacea*. We found that the molecular identity and the fates of early bryozoan blastomeres are similar to the putative homologous blastomeres in spiral-cleaving embryos.

**Conclusions:**

Our work suggests that bryozoans have retained traits of spiral development, such as the early embryonic fate map, despite the evolution of a novel cleavage geometry. These findings provide additional support that stereotypic cleavage patterns can be modified during evolution without major changes to the molecular identity and fate of embryonic blastomeres.

**Electronic supplementary material:**

The online version of this article (doi:10.1186/s12915-017-0371-9) contains supplementary material, which is available to authorized users.

## Background

Cleavage is the sequence of cell divisions that turns a zygote into a multicellular embryo, and plays an essential role in the specification of cell fates before the onset of gastrulation. A cleavage pattern can be variable, where the blastomere positions are not predictable (e.g., mouse), or stereotypic (e.g., ascidian), where the embryonic cell divisions form a precise, identifiable three-dimensional pattern of blastomeres [1]. There is evidence that different types of cleavage can dictate different underlying mechanisms of cell fate specification [1]; however, it is still largely unknown how a stereotypic cleavage pattern affects the evolution of animal morphology [2]. Cleavage patterns are highly diverse, they can even differ between closely related species [3, 4], or remain conserved in different animal lineages over long evolutionary periods [5]. A notable example of the latter is known as *spiral cleavage*, and is a rich framework to investigate the relation between development and evolution.

Spiral cleavage occurs in molluscs, annelids, nemerteans, and polyclad flatworms [6–16]. In these groups, the fertilized eggs divide through a highly stereotypic cleavage pattern where blastomeres at the 4-cell stage cleave with the mitotic spindles oblique to the animal–vegetal axis, alternating direction (clockwise and counterclockwise) at each division cycle, termed the spiral cleavage pattern [5, 6, 17–19]. This determinate developmental mode allowed for the identification of homologous blastomeres across taxa and unprecedented detail in the comparison of animal embryogenesis, further revealing that spiral-cleaving embryos not only have the same cleavage pattern, but homologous blastomeres between groups have a similar fate in the larval and adult tissues [5, 18]. The study of spiral cleavage thus revealed that, in contrast to late developmental stages, early development can remain conserved for extended evolutionary periods, shaping our current understanding about the relation between ontogeny and phylogeny [20–24].

Even though spiral cleavage has been modified in a multitude of ways throughout evolution with changes in blastomere sizes and cell fate specification [5, 17–19], the cleavage pattern itself remained fairly conserved. Known cases where the spiral cleavage pattern was lost is usually associated with drastic developmental changes, such as the transition to a syncytial blastoderm in cephalopods [25], or the evolution of extra-embryonic yolk cells in platyhelminthes [26]. However, the recent improvements in the resolution of protostome relationships revealed that the spiral cleavage pattern has been drastically modified or lost in even more groups than previously thought [5].

**Fig. 1:**
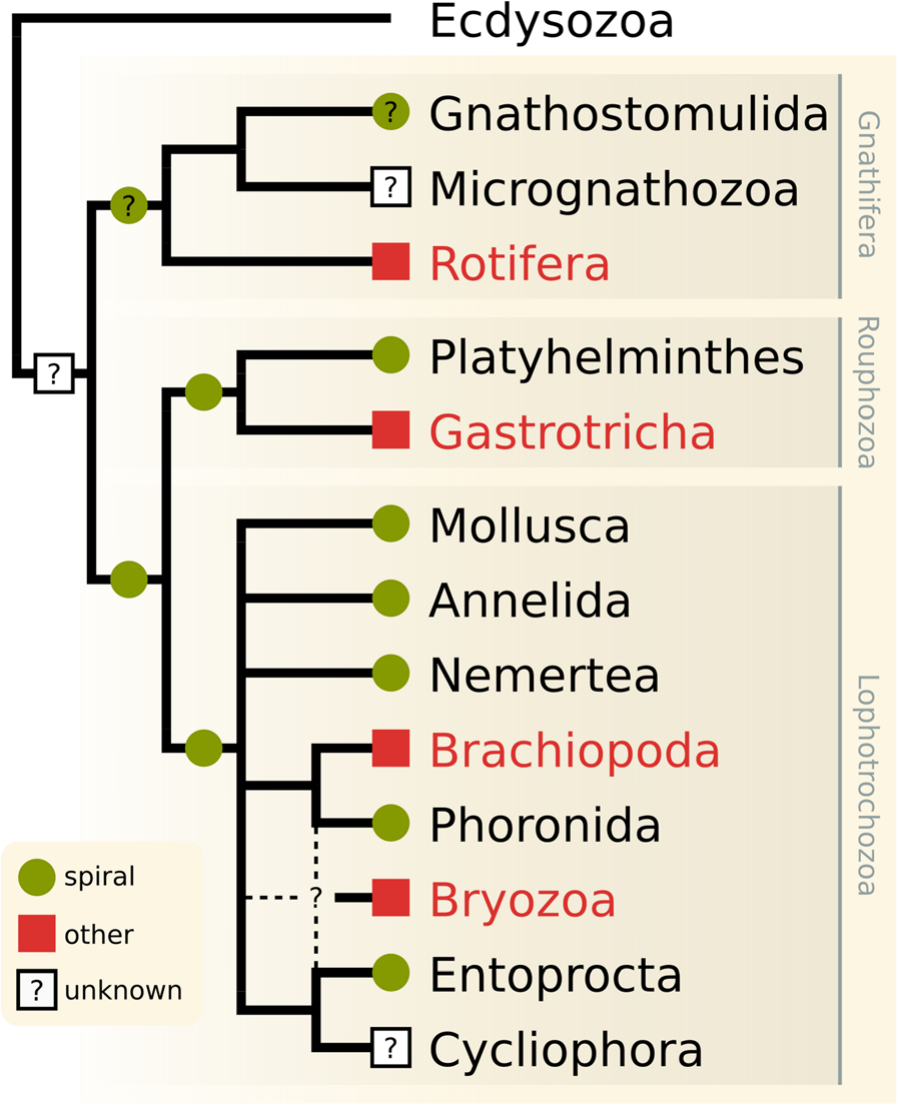
Phylogenetic distribution of the spiral cleavage geometry in the Spiralia. Green circles indicate taxa that exhibit a spiral cleavage geometry (alternating oblique cell divisions). Red squares indicate groups whose cleavage geometry is not spiral. White squares with question mark indicate taxa where the cleavage geometry is unknown. The nodes mark the presumed ancestral cleavage pattern of the branch. Green circles with question mark indicate preliminary, but not conclusive evidence of a spiral cleavage geometry in Gnathostomulida [183]. Spiralian relationships based on [27, 29–32], and cleavage data based on [5] (most clades), [184] (Phoronida), [185, 186] (Entoprocta), [33] (Bryozoa), [34] (Brachiopoda), [35] (Gastrotricha), and [36] (Rotifera). Dashed lines indicate alternative placements for Bryozoa

Spiral cleavage is a synapomorphy for the Spiralia (Lophotrochozoa *sensu lato*, after [27]), a major protostome clade containing all spiral-cleaving groups [28]. However, not all spiralians (i.e., animals belonging to the clade Spiralia) display a spiral cleavage pattern during embryogenesis. Recent spiralian phylogenies [27–32] indicate that clades that do not exhibit oblique cell divisions, such as the bryozoans [33], brachiopods [34], gastrotrichs [35], and rotifers [36], must have modified or lost the ancestral spiral cleavage pattern during evolution [5, 37] (Fig. 1). For this reason, such groups are essential to understand how cleavage patterns and blastomere fates evolve and can uniquely reveal which developmental traits, if any, remained conserved in the evolutionary transition from spiral to a derived cleavage geometry.

In the current work, we investigate the development of a group that lost the spiral cleavage pattern during evolution – the bryozoans. These sessile colonial invertebrates occur in oceans worldwide and have fairly diverse reproductive strategies and larval stages [38, 39], but none of the species investigated so far display a spiral arrangement of embryonic blastomeres [38]. Bryozoans display a unique stereotypic cleavage pattern with a biradial arrangement of the blastomeres that is widely conserved within the group.

Previous studies of bryozoan embryology [40–45] suggest that the animal-most blastomeres give rise to the apical disc and aboral epithelium of the larva, the vegetal-most derivatives of the animal blastomeres form the ciliated band, and the vegetal blastomeres produce the oral epithelium and endomesoderm [38, 39, 46]. This coarse fate map appears to be overall similar to that of spiral-cleaving embryos [47, 48]. However, cleavage patterns have only been systematically followed until the 64-cell stage [43, 45] and, as of today, there is no detailed cell lineage or fate map of a bryozoan larva. Several basic developmental questions remain unsolved. For example, the relation between the embryonic animal–vegetal axis and the larval body axes is unclear [48], and the fate of the blastopore remains to be confirmed [39, 41, 44, 49]. Finally, the fate of internalized cells has not been traced [39] and the source of mesoderm remains an especially contentious topic [49].

In this study, we investigate the embryogenesis of the cosmopolitan gymnolaemate species *Membranipora membranacea* (Linnaeus, 1767) to understand the evolutionary transition from a spiral to a biradial cleavage pattern. We take advantage of the vast cell lineage data available for spiralians and the growing literature on spiralian gene expression, to compare the molecular identity and fate of embryonic blastomeres between the bryozoan and other spiral-cleaving embryos with cellular resolution. We were able to identify the embryonic source of most larval tissues of *M. membranacea* based on 4D microscopy recordings, and to combine this cell lineage data with the activity of the MAPK pathway and expression of several conserved developmental markers, generating a detailed overview of the blastomere identities and fates in the bryozoan. The comparison to a typical spiral development reveals that the early blastomeres of *M. membranacea* share similar molecular identities and fates with other spiral-cleaving embryos, despite the contrasting cleavage pattern. Given the phylogenetic position of bryozoans, we suggest these coincident developmental traits were inherited from a spiral-cleaving ancestor during the evolutionary transition from spiral to biradial cleavage. The findings support the hypothesis that stereotypic cleavage patterns can be modified during evolution without major changes to blastomere gene expression and fates. Our study highlights the power of the comparative approach to address fundamental questions of development and evolution, such as the relation between cleavage patterns and fate maps.

## Results

### General development and data overview

**Fig. 2:**
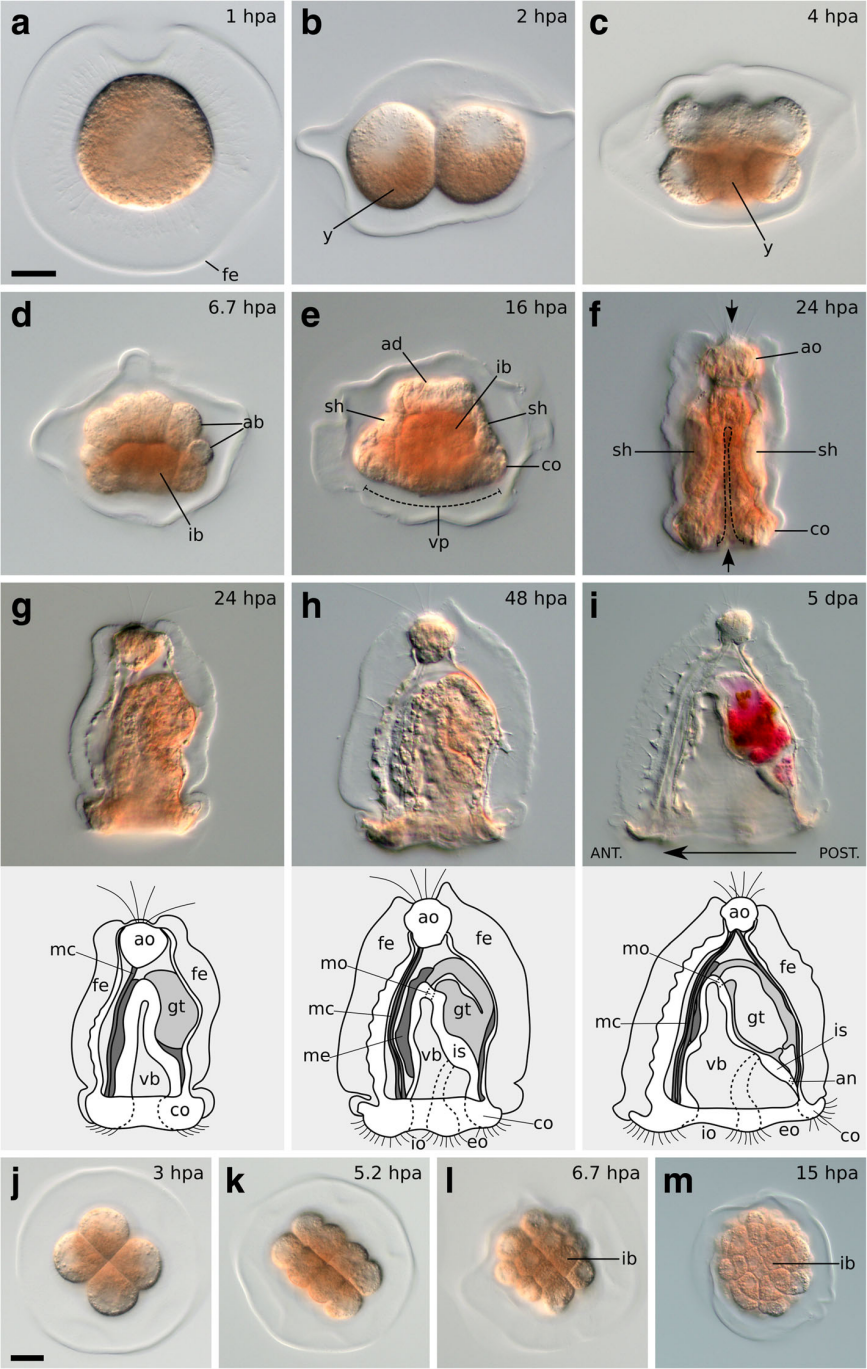
Overview of *M. membranacea* development. **a** Vegetal view of an activated egg becoming spherical (1 hpa). **b**–**i** Animal pole is *top* and vegetal pole is *bottom*. **b** 2-cell stage showing higher amount of yolk (*y*) on the vegetal side (2 hpa). **c** 8-cell stage with yolk positioned on the inner cytoplasmic portions (4 hpa). **d** 28-cell stage (6.7 hpa). Large vegetal blastomeres (*ib*) carry most of the yolk (yellowish color), while less yolk is present in the animal blastomeres (*ab*). **e** Anterior view of a mid gastrula stage (16 hpa) with a prominent apical disc (*ad*), shell primordia (*sh*), coronal cells (*co*), the vegetal ectodermal plate (*vp*) demarcated by a dashed line, and the internalized yolk-rich blastomeres (*ib*). **f** Frontal view of a late gastrula stage (24 hpa) after the vegetal ectoderm invaginated (*dashed line*) and the embryo extended in the animal–vegetal axis. Larval structures begin to be functional by this stage, including the apical organ (*ao*), shell valves (*sh*), and corona (*co*). The cilia of the apical tuft and coronal cells break through the fertilization envelope (*arrows*) at this stage. **g**–**i** Lateral views of the cyphonautes morphogenesis with the larval structures illustrated below each panel. The anteroposterior axis is labeled according to [51]. **g** A late gastrula stage (24 hpa), (**h**) an early larva (48 hpa), and (**i**) a fully functional cyphonautes larva 5 days post activation (dpa). Its gut is filled with the red microalgae *Rhodomonas* sp, which we add to the cultures as a food source. **j**–**m** Vegetal views showing beginning of gastrulation. **j** 8-cell stage (3 hpa). **k** 16-cell stage (5.2 hpa). **l** 28-cell stage (6.7 hpa) with four central vegetal blastomeres (*ib*). **m** 90-cell stage with vegetal blastomeres (*ib*) being internalized (15 hpa). *ab* animal blastomeres, *ad* apical disc, *an* anus, *co* corona, *eo* exhalant opening, *fe* fertilization envelope, *gt* gut, *ib* inner vegetal blastomeres, *io* inhalant opening, *is* internal sac, *mc* muscle cell, *me* mesodermal tissue, *mo* mouth, *sh* shell primordium, *vb* vestibule, *y* yolk. Scale bars = 20 μm

Colonies of *M. membranacea* spawn fertilized discoidal eggs into the water column [50]. The released eggs undergo activation, quickly become spherical (Fig. 2a), and initiate cleavage at around 2 hours post activation (hpa) with a discernible accumulation of yolk at the vegetal pole (Fig. 2b). Throughout development, the embryo maintains close contact with the fertilization envelope via abundant cytoplasmic extensions (Fig. 2a, i). The yolky cells at the vegetal pole are internalized during gastrulation (Fig. 2c–e, j–m) and, by the mid gastrula stage (16 hpa), the primordia of the apical organ (apical disc) and of the ciliated band (corona) are visible (Fig. 2e). The vegetal plate invaginates and the embryo elongates along the animal–vegetal axis forming a late gastrula at 24 hpa with clearly defined larval structures (i.e., apical organ, shell, gut, and corona) (Fig. 2f, g). At this point the fertilization envelope opens at the animal and vegetal ends and the embryo begins to swim by ciliary beating (Fig. 2f). The internal cavity (vestibule) widens in the anteroposterior axis resulting in the typical laterally compressed, triangular shaped and shelled feeding larva of gymnolaemate bryozoans – the cyphonautes (Fig. 2h, i) [51].

In this study, we recorded four individual embryos from the 2-cell stage at 2 hpa (Fig. 2b) until the late gastrula stage at 24 hpa (Fig. 2f, g). Due to the opaqueness of the embryo, it was necessary to trace animal and vegetal clone populations in different individuals, namely “wild type 1” (wt1) and wt2, respectively (Additional file 1: Video S1). To evaluate the potential variability between individual embryos, we recorded two additional animal pole views (embryos wt3 and wt4, Additional file 2: Video S2). We were able to trace a greater number of individual cells until the mid gastrula stage at 16 hpa (Additional file 3: Figure S1), when the primordia of most larval structures were clearly defined and the fate of the cells could be determined (Fig. 2e).


Additional file 1: Video S1. 4D recordings of *M. membranacea* embryos wt1 and wt2. Embryo wt1 was recorded from the animal pole (*left*) and wt2 was recorded from the vegetal pole (*right*). We focused the different focal planes of each time point into a single image (focus stacking) to reveal the overall embryo morphology. The time-lapse stack-focused images were animated at 25 frames per second (1000× acceleration) and the developmental stages of both embryos were synchronized by accelerating the video of wt2 by 10% in relation to wt1. Duration = 21.8 h (from ~3 hpa to ~25 hpa). (MP4 19279 kb)
Additional file 2: Video S2. 4D recordings of *M. membranacea* embryos wt3 and wt4. Embryos wt3 (*left*) and wt4 (*right*) were recorded from the animal pole. We focused different focal planes of each time point into a single image (focus stacking) to reveal the overall embryo morphology. The time-lapse stack-focused images were animated at 25 frames per second (1000× acceleration) and the developmental stages of both embryos were synchronized by accelerating the video of wt3 by 5% in relation to wt4. Duration = 23.0 h (from ~3 hpa to ~25 hpa). (MP4 19389 kb)


Overall, the data we collected suggests the cell lineage of individual *M. membranacea* embryos is highly stereotypic and exhibits small variation in the timing of cell divisions (see below). However, due to our limited sample size, we cannot fully account for the cell fate variability that might exist in bryozoan development, particularly at later stages. The results we report below thus reflect the consensus data between the four *M. membranacea* embryos tracked in this study.

### Cleavage pattern and embryonic axes

**Fig. 3:**
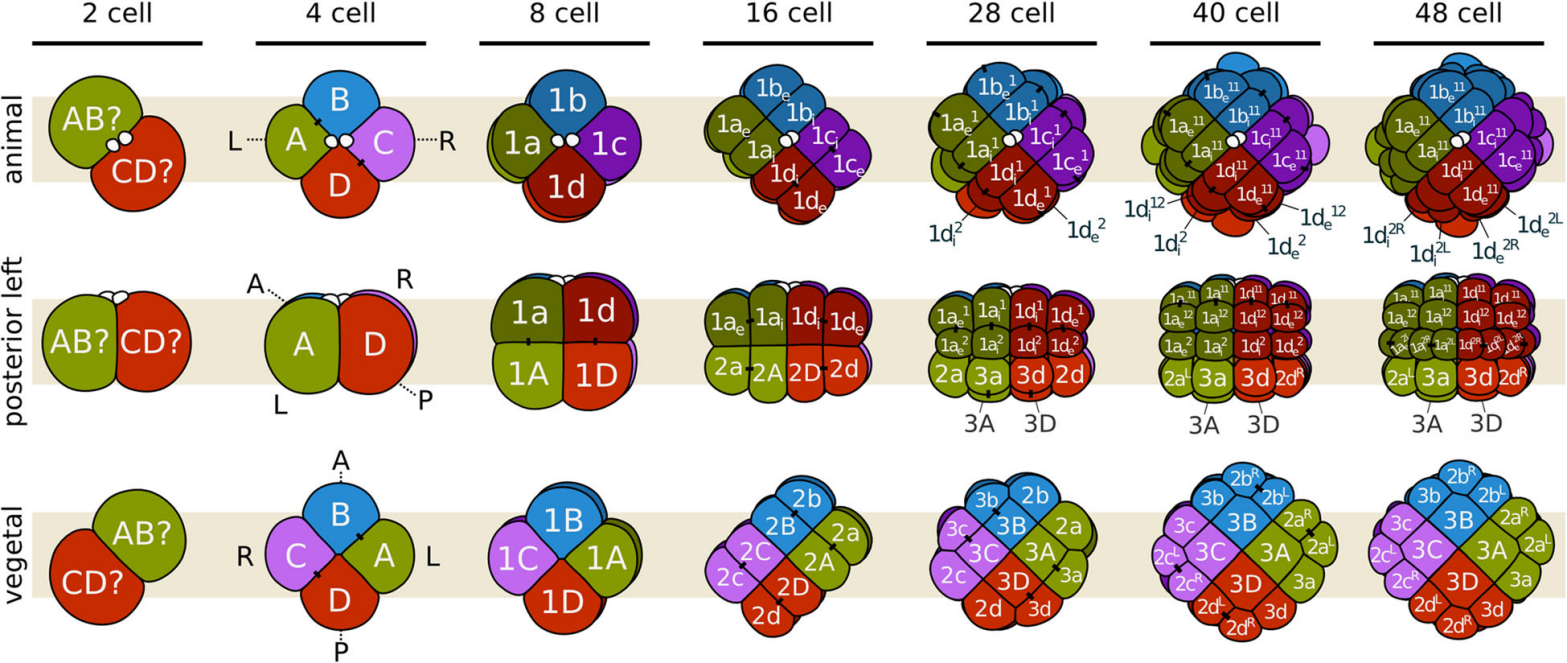
Cleavage pattern and orientation of the embryonic axes of *M. membranacea*. Quadrant identity was determined backwards from 4D microscopy recordings and we do not know if it is determined before the 16-cell stage. The nomenclature was adapted from the spiral cleavage notation to describe the peculiarities of bryozoan cleavage. See Methods for details

**Fig. 4:**
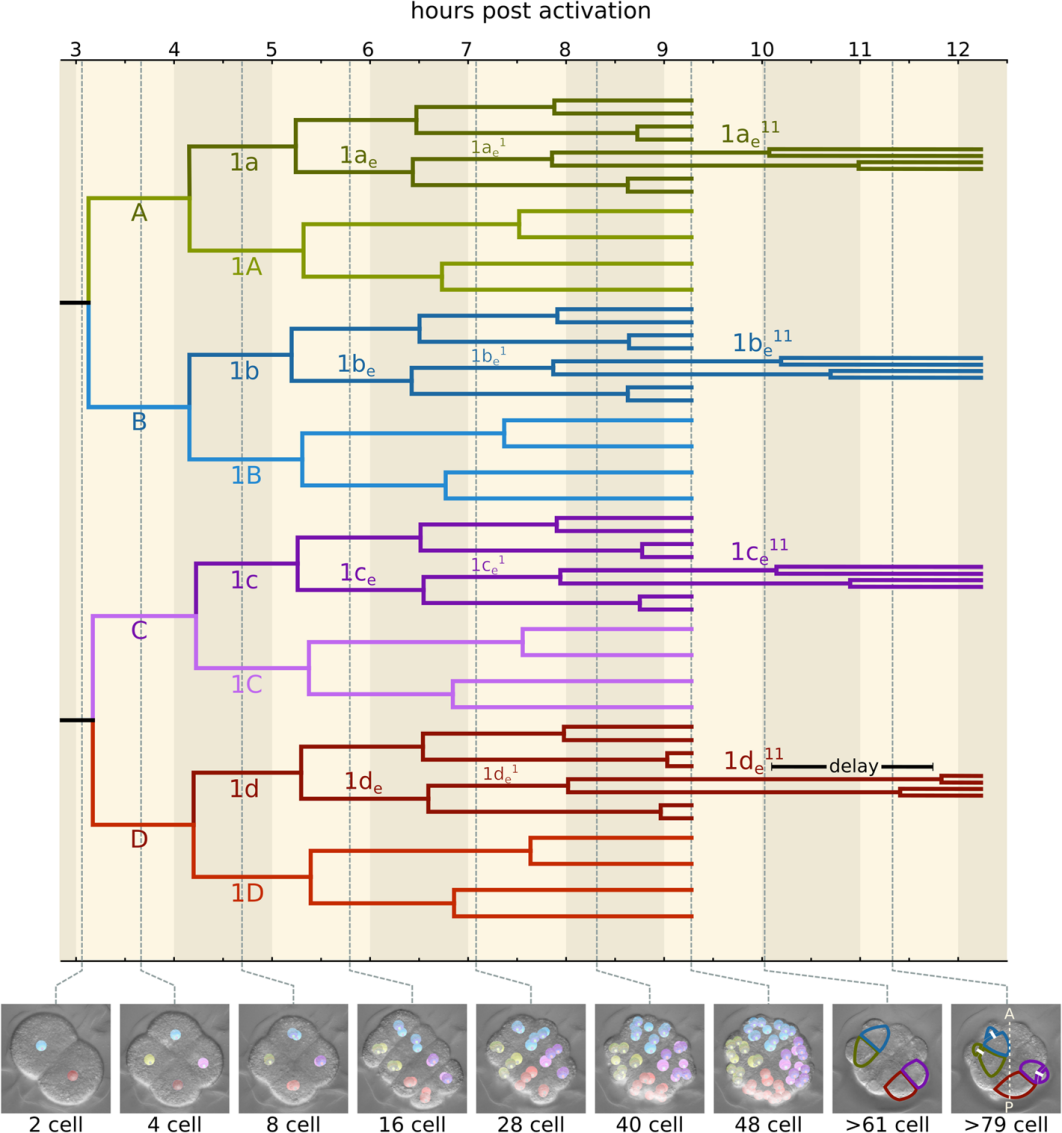
Timed cell lineage of *M. membranacea* and the break of biradial symmetry. Panels on the bottom show the developmental stages with the cell tracing overlay until 48 cells. The outlines in the last two panels (>61 and > 79 cells) indicate the cells 1a_e_^11^–1d_e_^11^, and the prominent delay in the division of 1d_e_^11^. The anteroposterior axis is denoted by a dashed line in the last panel. Quadrant color coding: A (green), B (blue), C (purple), D (red)

**Fig. 5:**
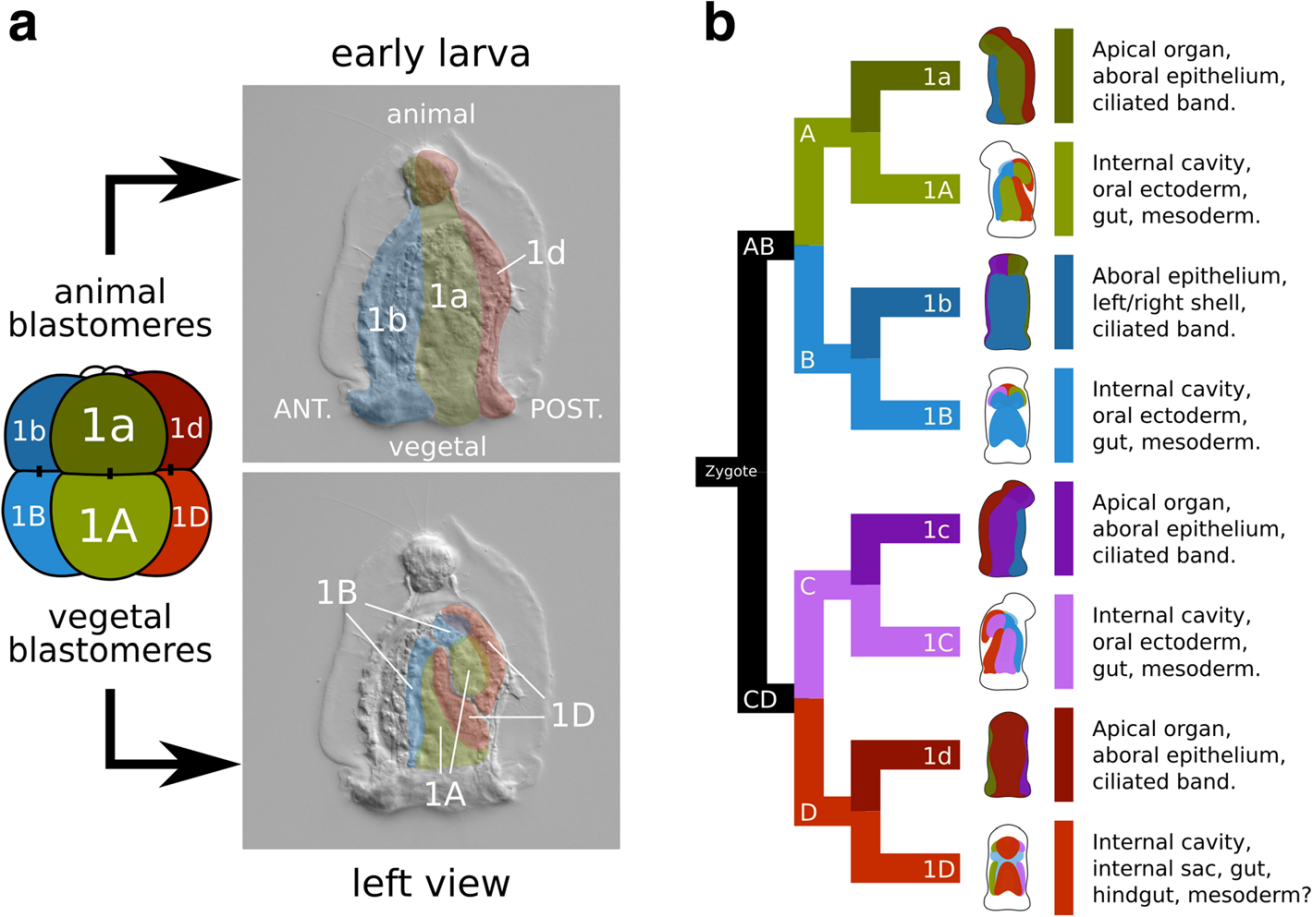
Larval fates of *M. membranacea* 8-cell stage blastomeres. **a** Illustration based on the cell lineage data representing the overall fates of the animal (1a–1d) and vegetal (1A–1D) blastomeres. Animal blastomeres give rise to the apical organ, aboral epithelium and corona. Vegetal blastomeres give rise to the vestibule epithelium, oral ectoderm, mesoderm, and gut. **b** Larval structures derived from each of the eight blastomeres. Quadrant color coding: A (green), B (blue), C (purple), D (red)

The cleavage of *M. membranacea* is biradial as previously described for gymnolaemates (Fig. 3) [38, 39, 48, 49, 52]. At 15 °C, the first cell division occurs between 1 and 2 hpa and produces two equal blastomeres with a meridional cleavage furrow. The second division is also meridional and perpendicular to the first, resulting in four blastomeres of equal sizes around 3 hpa (Fig. 2j; Fig. 3; Fig. 4, 4-cell). We labeled the blastomere that gives rise to the posterior structures of the larval body as “D” (see Fig. 5 for fate map overview and “Methods” for nomenclature details). In most embryos, the cell sister of the D blastomere gives rise to the right side of the embryo [53]. At 4 hpa, an equatorial third division gives rise to four animal blastomeres with lower yolk content (1a–1d), and four equally sized vegetal blastomeres with a greater amount of yolk displaced towards the center of the embryo (1A–1D) (Fig. 2c; Fig. 3; Fig. 4, 8-cell). During the next division at 5.2 hpa, each animal blastomere divides meridionally, parallel to the plane of the first cleavage, forming a 16-cell stage embryo that clearly differs from the canonical spiral cleavage pattern (Fig. 2k; Fig. 3; Fig. 4, 16-cell). Since these blastomeres occupy the same position along the animal–vegetal axis, and thus cannot be objectively labeled with superscript ^1^ or ^2^, they received the subscript _i_ or _e_ to indicate their internal or external position in relation to the central axis of the embryo (see “Methods” for nomenclature details). The vegetal blastomeres cleave in the same manner, but slightly after. At the 16-cell stage (5.2 hpa), yolk-rich cells (2A–2D) lie inner to the outer vegetal cells of the second quartet (2a–2d) and the embryo is clearly biradial.

During subsequent stages, the eight animal blastomeres of *M. membranacea* act as octets, dividing synchronously (Fig. 3). The first octet (animal pole cells 1q, four inner and four outer cells) divides equatorially making a brief 24-cell stage and the octets 1q^1^ and 1q^2^ (6.5 hpa). This division is shortly followed by an unequal cleavage originating the third quartet (3a–3d) from the four inner vegetal blastomeres at 6.7 hpa (Fig. 2l; Fig. 3; Fig. 4, 28-cell). Outer vegetal cells of the second quartet (2q) divide parallel to the second division at 7.5 hpa, resulting in 12 outer vegetal cells (3a–3d, 2a^R^–2d^R^, 2a^L^–2d^L^) that surround four large blastomeres in the vegetal plate (3A–3D) at the 32-cell stage. At 8 hpa, the top animal octet (1q^1^) divides, forming a 40-cell embryo (Fig. 3; Fig. 4, 40-cell). Finally, the vegetal most animal octet (1q^2^) divides meridionally at 8.6–9 hpa forming an equatorial row of cells above the vegetal blastomeres (Fig. 3; Fig. 4, 48-cell).

### Cell lineage variability

We found little variation between the cell lineages of the four embryos. That is, a particular cell in one embryo has the same lineage history, occupies the same relative position and divides roughly at the same time as the respective cell in a different embryo. A direct comparison between the four *M. membranacea* embryos reveals that the lineages overlap well, exhibiting only small variations in the timing of cell divisions (Additional file 4: Figure S2A). We quantified this variability by plotting the time of birth of individual cells and calculating the magnitude of variation across embryos (Additional file 4: Figure S2A). The timing is fairly consistent until 9 hpa and homologous cells in different embryos divide less than 20 min apart from each other (Additional file 4: Figure S2B). We also found that embryos wt1, wt3, and wt4 have similar timing, with cell divisions occurring within 10 min of each other (Additional file 4: Figure S2C). These data suggest the development of *M. membranacea* is highly stereotypical with consistently timed cell divisions between individuals.

Within a single embryo, the cell divisions between the correspondent blastomeres of each quartet are mostly synchronous up to the 64-cell stage at 11 hpa (Fig. 4). At this point, we observe the first significant asynchronies in the cell divisions of a quartet, both occurring in the posterior D quadrant. The cell 1d_e_^11^ divides 2 h later than its quartet correspondents 1a_e_^11^, 1b_e_^11^, and 1c_e_^11^ (Fig. 4 and Additional file 5: Video S3), while the cell 1d_i_^12^ divides approximately 1 h before its partners 1a_i_^12^, 1b_i_^12^, and 1c_i_^12^ (Additional file 6: Figure S3). We also observe, in the four embryos, a 3.5 h delay in the division of 3D, relative to the divisions of 3A–3C. These D quadrant asynchrony events occur with surprising consistency between the different bryozoan embryos at least until the stages analyzed in this study. We could detect a few cases of variability in the timing of divisions, but overall our data indicates the development of *M. membranacea* varies little between individuals.


Additional file 5: Video S3. Delayed D quadrant cell division in *M. membranacea*. Development of embryo wt1 (left) and wt3 (right) annotated with cell models from Simi BioCell [168]. The lineage of the quartet 1q_e_^11^ is highlighted in color from the 4-cell stage (3 hpa). The cell 1d_e_^11^ (red) divides 2 h later than its quartet correspondents 1a_e_^11^ (green), 1b_e_^11^ (blue), and 1c_e_^11^ (purple). This delayed division is one of the first morphological manifestations of the break in biradial symmetry and the establishment of the anteroposterior axis of the embryo. (MP4 18789 kb)


Finally, at a similar time point, we observe the first difference in the orientation of the cleavage plane between quartet cells. While 1d_e_^12^ divides equatorially, 1a_e_^12^, 1b_e_^12^, and 1c_e_^12^ divide meridionally. The asynchrony in the D quadrant and shift in cleavage orientation are the first morphological events that mark the break in the biradial symmetry of the bryozoan embryo.

### Cellular origin of larval tissues

The larval body of *M. membranacea* develops from the four quadrants in a symmetrical manner, each lineage contributing almost equally to the structures on their respective sides: D = posterior, C = right, B = anterior, and A = left (Fig. 5a and Additional file 7: Video S4).


Additional file 7: Video S4. Cells tracked in the embryo wt1 color-coded by quadrant of origin. Video is the same as Additional file 1: Video S1 but annotated with a maximum projection of the cell models from MaMuT [169]. Cells out of the focus plane are also shown. (MP4 19238 kb)


**Fig. 6:**
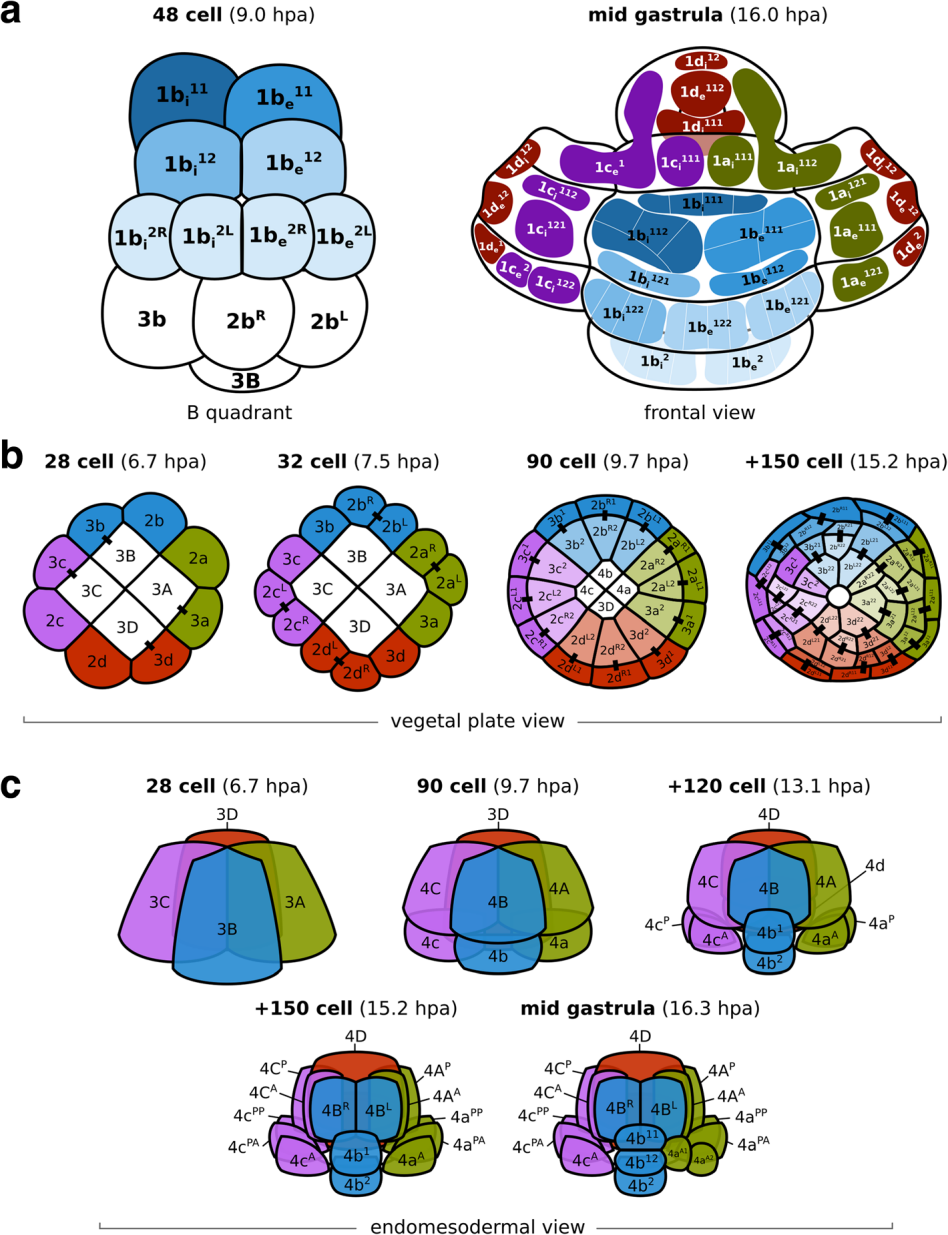
Details of the fate map and cleavage pattern of the animal, vegetal, and internalized blastomeres of *M. membranacea*. **a** Representation of the B quadrant at the 48-cell stage (9.0 hpa) in frontal view (*left*). Frontal view of the embryo at the mid gastrula stage (16.0 hpa) with left, right, and top regions “opened” for visualization (*right*); the shades of blue correspond to the blastomeres of the 48-cell stage (*left*). White lines illustrate cell borders of further progeny from the blastomeres indicated. Color-coding of the remaining blastomeres indicate their quadrant of origin. **b** Cleavage patterns of the vegetal ectoderm viewed from the vegetal pole at the 28-cell stage (6.7 hpa). The vegetal plate progenitors consist of 12 blastomeres lining at the 32-cell stage (7.5 hpa). These cells divide once, forming 12 derivatives, lining the forming blastopore at the 90-cell stage (9.7 hpa). At the subsequent divisions (15.2 hpa), progeny from the cells at the vertices (2a^L2^, 2b^R2^, 2d^R2^, and 2c^L2^) disconnect from the blastopore lip. At this stage, only eight cells are lining the blastopore. The cells 3c^1^ and 3c^2^ do not divide. **c** Cleavage patterns of the four large vegetal blastomeres internalized during gastrulation, frontal view. Quadrant color coding: A (green), B (blue), C (purple), D (red)

Progeny of the first quartet of animal blastomeres (1a–1d) gives rise to animal ectodermal structures such as the apical organ, the aboral epithelia and the corona (Fig. 5a). The apical organ is derived from derivatives of the apical-most cells 1a^1^, 1c^1^, and 1d^1^ (Figs. 5b and 6a). Cells 1a and 1c form the lateral and anterior most portion of the apical organ while the posterior cell 1d contributes not only to the posterior portion, but also to the tissues at the base of the apical organ (Fig. 6a). Thus, the cell 1b is the only blastomere of the first animal quartet that does not contribute to the apical organ. Epithelial cells between the apical organ and the corona are mostly derived from the octets 1q^11^ and 1q^12^. Outer coronal cells originate from 1q^12^ and 1q^2^ while inner coronal cells (turned inwards after the invagination of the vegetal plate) are derived from 1q^2^ (Fig. 6a). For a detailed overview of cell fates see Additional file 8: Figure S4.

The vegetal blastomeres 1A–1D form the epithelium of the vestibule, the oral/anal ectoderm, as well as the cells internalized during gastrulation, which originate the endoderm and mesoderm of the cyphonautes larva (Fig. 5a and Additional file 9: Video S5). The cellular arrangement at the vegetal plate in a 32-cell embryo (7.5 hpa) consists of 12 outer cells (3a–3d, 2a^R/L^–2d^R/L^) and four large inner blastomeres (3A–3D) (Fig. 6b and Additional file 10: Figure S5B). Here, we define gastrulation as the internalization of these four vegetal cells. It occurs by delamination and epiboly in two rounds of division of the outer vegetal 12-tets, which divide radially, pushing the four larger blastomeres internally and outlining a blastopore (Fig. 6b and Additional file 10: Figure S5B–F). At the 90-cell stage (9.7 hpa), 12 cells define the blastopore lip, but this number gets reduced to 8 cells after the next division (Fig. 6b and Additional file 10: Figure S5F–I). From the 12 vegetal cells, one does not divide (3c^2^) and continues to line the right side of the blastopore lip (Fig. 6b and Additional file 10: Figure S5E). Cells at the vertices of the blastopore at the 90-cell stage at 9.7 hpa (2a^L2^, 2b^R2^, 2c^L2^, and 2d^R2^) get pushed away from the blastopore lip, which now consists of 8 cells (Fig. 6b and Additional file 10: Figure S5I). Blastomeres not forming the blastoporal lip also undergo the same round of two radial divisions except for 3c^1^, the sister of 3c^2^. The derivatives of these 12 vegetal outer blastomeres form the whole ectoderm that invaginates and develops into the epithelia of the vestibule and preoral funnel. Thus, in the course of the invagination of the vegetal plate, and of the animal–vegetal elongation of the embryo, the blastopore in *M. membranacea* becomes the larval mouth.


Additional file 9: Video S5. Cells tracked in the vegetal ectoderm of embryo wt2 color-coded by generation. Video is the same as Additional file 1: Video S1 but the embryo is rotated 180°. The angle of the embryo was also slightly adjusted in the BigDataViewer [170] to align the vegetal surface and only a few slices of the z-axis are shown. Cell models were exported from MaMuT [169]. Video shows the internalization of the large vegetal blastomeres (black) and the rounds of cell divisions of the vegetal 12-tets (blue). See Additional file 10: Figure S5 for a detailed view of the events in the vegetal ectoderm. (MP4 19150 kb)


**Fig. 7:**
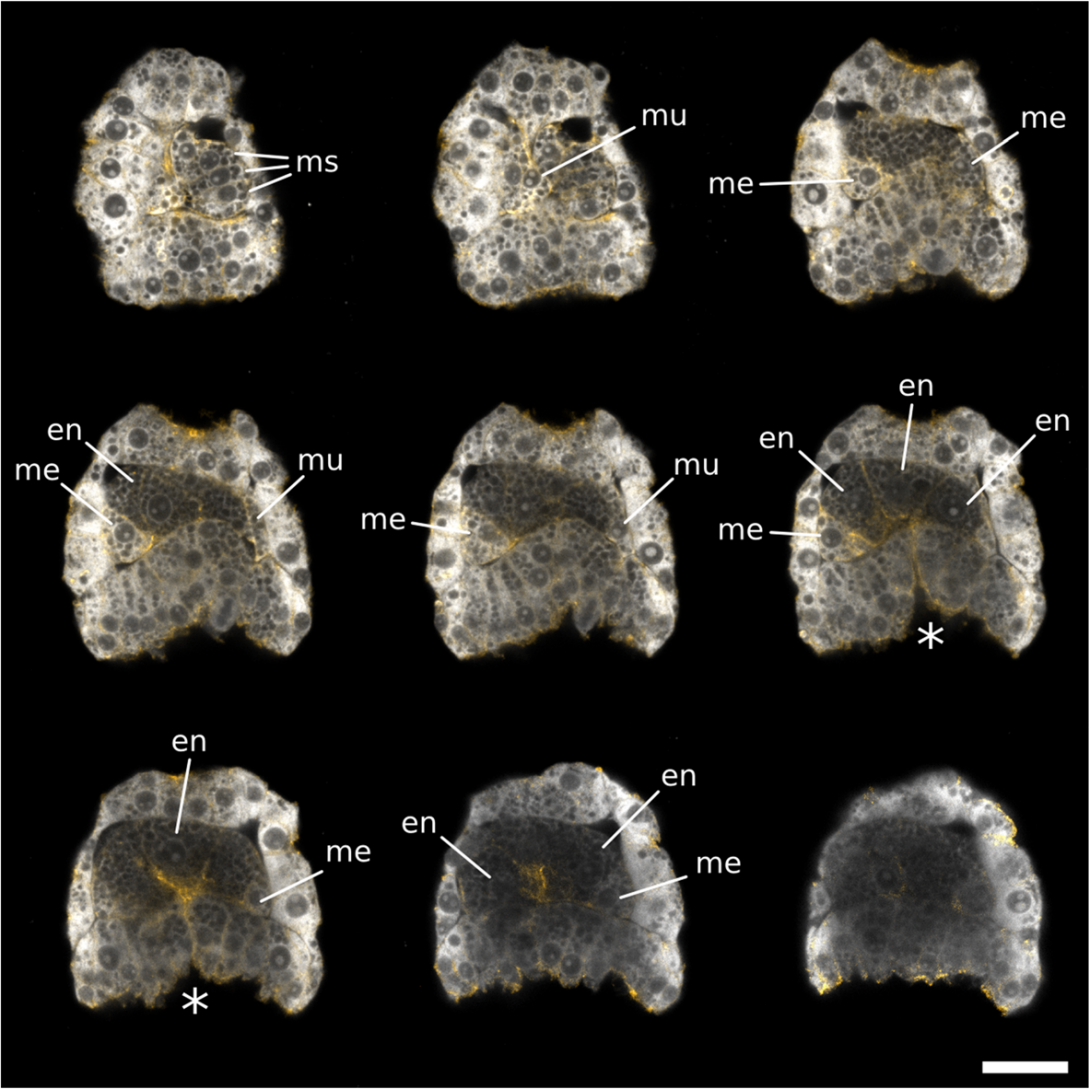
Mesodermal and endodermal cells in *M. membranacea*. Maximum intensity projections of 2–3 slices from one confocal stack at the mid gastrula stage. View of the anterior/right side of the embryo (*top-left*) to the posterior/left side (*bottom-right*). Samples stained with propidium iodide for DNA/RNA (*grays*) and with BODIPY FL phallacidin for f-actin (*orange*). *en* endodermal cells, *ms* mesodermal stack of cells from the B quadrant (4b derivatives), *mu* muscle cells reaching the apical organ, *me* other mesodermal cells. Asterisk indicates the blastopore. Scale bar = 20 μm


During epiboly (9.7 hpa, around 90 cells), three of the internalized large blastomeres (3A–3C) undergo a round of unequal division forming the basal cells 4a–4c and the apical cells 1A–1C (Fig. 6c). The division of the 3D cell occurs with a 3.5-h delay in comparison to the other blastomeres (13.1 hpa). This round of division sets apart the endoderm (4A–4D) from the mesodermal tissues (4a–4c) of *M. membranacea* cyphonautes larva. The cell 4d is also formed, but we could not resolve its fate. The cells 4a and 4c divide twice anteroposteriorly, forming a pair of lateral rows of mesodermal cells (Fig. 6c). The most anterior cells (4a^A^ and 4c^A^) form a bilateral pair of muscle cells extending from the corona to the apical organ (Fig. 7). Interestingly, one anterolateral cell (4a^A1^) migrates from the corona level until the apical organ during animal–vegetal elongation of the embryo (Additional file 11: Video S6). At the frontal portion of the larva, the cell 4b divides forming a column of cells stacking from the corona until the apical organ; the identity or role of these cells is unknown (Fig. 6c). Blastomeres 4A and 4C undergo anteroposterior divisions while 4B divides meridionally at 15.2 hpa lining up with the blastoporal opening and forming the endodermal tissues of the cyphonautes larva (Figs. 6c and 7).

Additional file 11: Video S6. Migration of a mesodermal cell in the embryo wt1. The anterolateral cell 4a^A1^ (*dashed circle*) migrates from the vegetal region near the corona to the animal region near the apical organ, during animal–vegetal elongation of the embryo. This was the only cell migration event we were able to track. The video was exported using MaMuT’s “Extract track stack” action. (MP4 3485 kb)


### MAPK activity

**Fig. 8:**
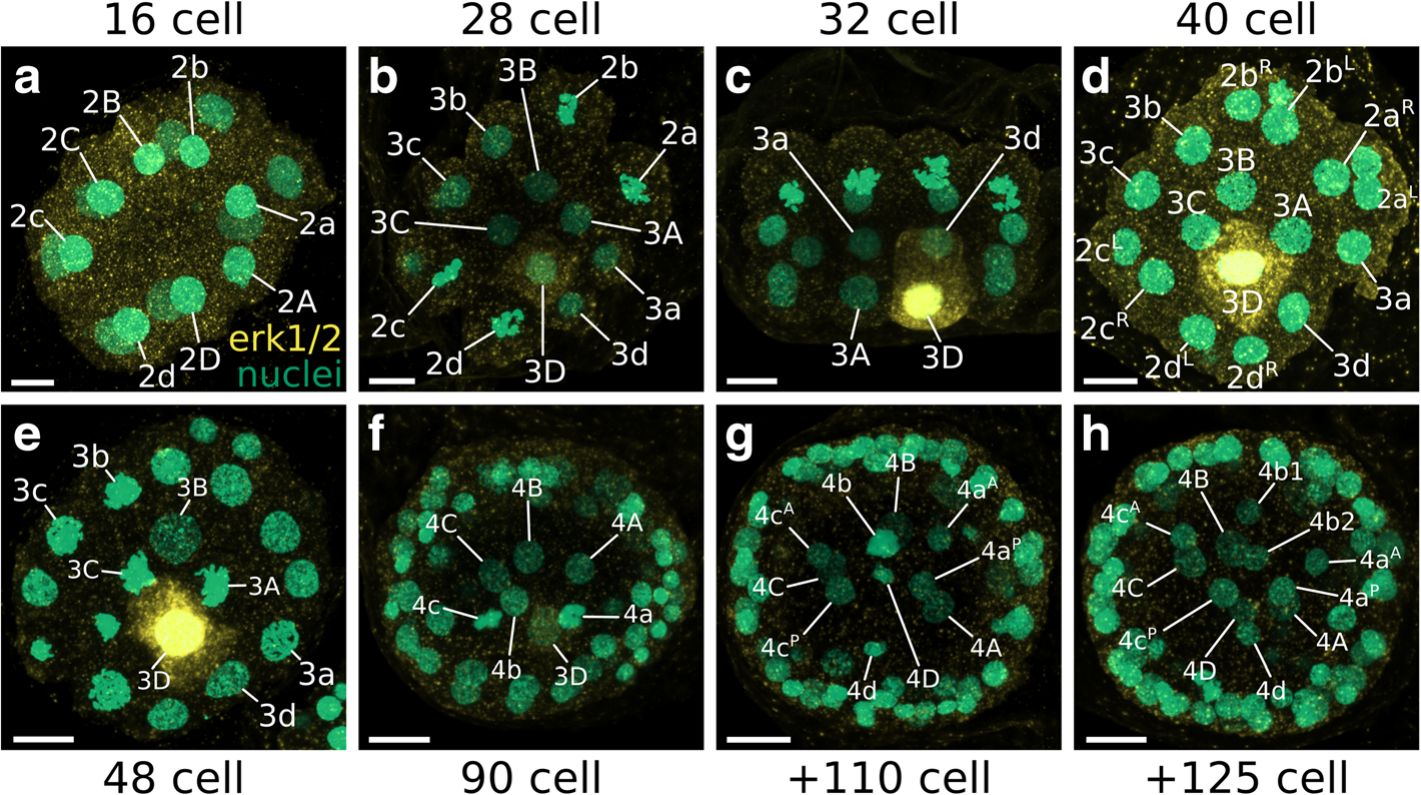
MAPK activity during the development of *M. membranacea*. Confocal maximum intensity projections of embryos incubated with the antibody against the activated form of MAPK (Diphosphorylated ERK-1&2) (*yellow*) and counterstained with Sytox Green for nuclei (*green*). **a** No detectable levels of activated MAPK at the 16-cell stage (5.2 hpa). **b** 28-cell stage in vegetal view at 6.7 hpa showing the first detectable MAPK activity. **c** Side view at 7.5 hpa showing the quadrants A and D of a 32-cell stage with activated MAPK in the cell 3D. **d** 40-cell stage at 8 hpa. **e** Vegetal view of a 48-cell stage at 9 hpa with blastomeres 3C and 3A undergoing mitosis. **f** Frontal view of an embryo with approximately 90 cells at 9.7 hpa. 3D cell shows a weaker signal for MAPK activity. **g** Embryo soon after the division of the 3D cell at 13.1 hpa. There are no detectable levels of MAPK activity in any cell. **h** Embryo with more than 125 cells (16 hpa) without any detectable levels of MAPK activity. Scale bar = 10 μm

Previous work revealed that the MAPK signaling pathway might establish the position of the dorsal organizer in molluscan embryos [54]. So far, all investigated molluscs show the asymmetric activation of MAPK in the 3D blastomere [54–57]. Using an antibody against the activated form of MAPK, we found that, in the bryozoan *M. membranacea*, the first detectable MAPK activity occurs in the 3D vegetal blastomere at the 28-cell stage (6.7 hpa) (Fig. 8b). MAPK activity persists in the 3D cell from the 28-cell to the 90-cell stage (9.7 hpa) and fades prior to the 3D division around 90-cell stage (Fig. 8b–f). MAPK activity is not continued in the progeny of 3D, 4D, or 4d (Fig. 8g–h) and was not detected in later embryonic stages.

### MAPK inhibition

The inhibition of the MAPK pathway in molluscs causes defects in the dorsoventral patterning [54–57] while, in annelids, MAPK-inhibited embryos have disorganized muscle and nerve tracts and overall shortened morphology [58–60]. We used the MEK inhibitor U0126 to investigate the role of the MAPK pathway in the development of *M. membranacea* at 10 °C.

We investigated the effects of different U0126 concentrations (1, 10, 25 μM) on the development of *M. membranacea* when applied at 2 hpa in the 2-cell stage (Additional file 12: Figure S6A). We found the severity of the phenotype correlates with the concentration of the inhibitor, where the higher concentrations of 10 and 25 μM result in the complete disruption of the normal morphology (Additional file 12: Figure S6A). These embryos show no identifiable larval structures, such as a differentiated apical organ or musculature, have a lower number of cells, and are shorter compared to control samples (Additional file 12: Figure S6B).

The proportion of embryos exhibiting a severe phenotype decreases when the treatment begins at later developmental stages (from 4–8 hpa, 8-cell), even though these time points precede the observed period of MAPK activity of *M. membranacea* (Additional file 13: Figure S7). Embryos treated from 10 hpa onwards show progressively milder phenotypes (Additional file 13: Figure S7). In treatments beginning at 10–16 hpa (16- to 90-cell), the larval structures, such as apical organ, ciliated band, and gut, are formed but the embryos are shorter and delayed in development in comparison to control embryos, while 18–24 hpa (early/mid gastrula) samples have almost normal morphology (Additional file 13: Figure S7).

Finally, to identify the developmental defects caused by the MEK inhibitor, we recorded *M. membranacea* embryos treated with 10 μM U0126 under the 4D microscope. We found the earliest abnormality associated with U0126-treated embryos is a misguided fourth cleavage (8–16 cell stage) in individuals exhibiting the severe phenotype, while embryos with milder phenotypes develop slower when compared to wild type, but do not show any obvious cleavage abnormalities (Additional file 14: Figure S8).

### Gene expression

**Fig. 9:**
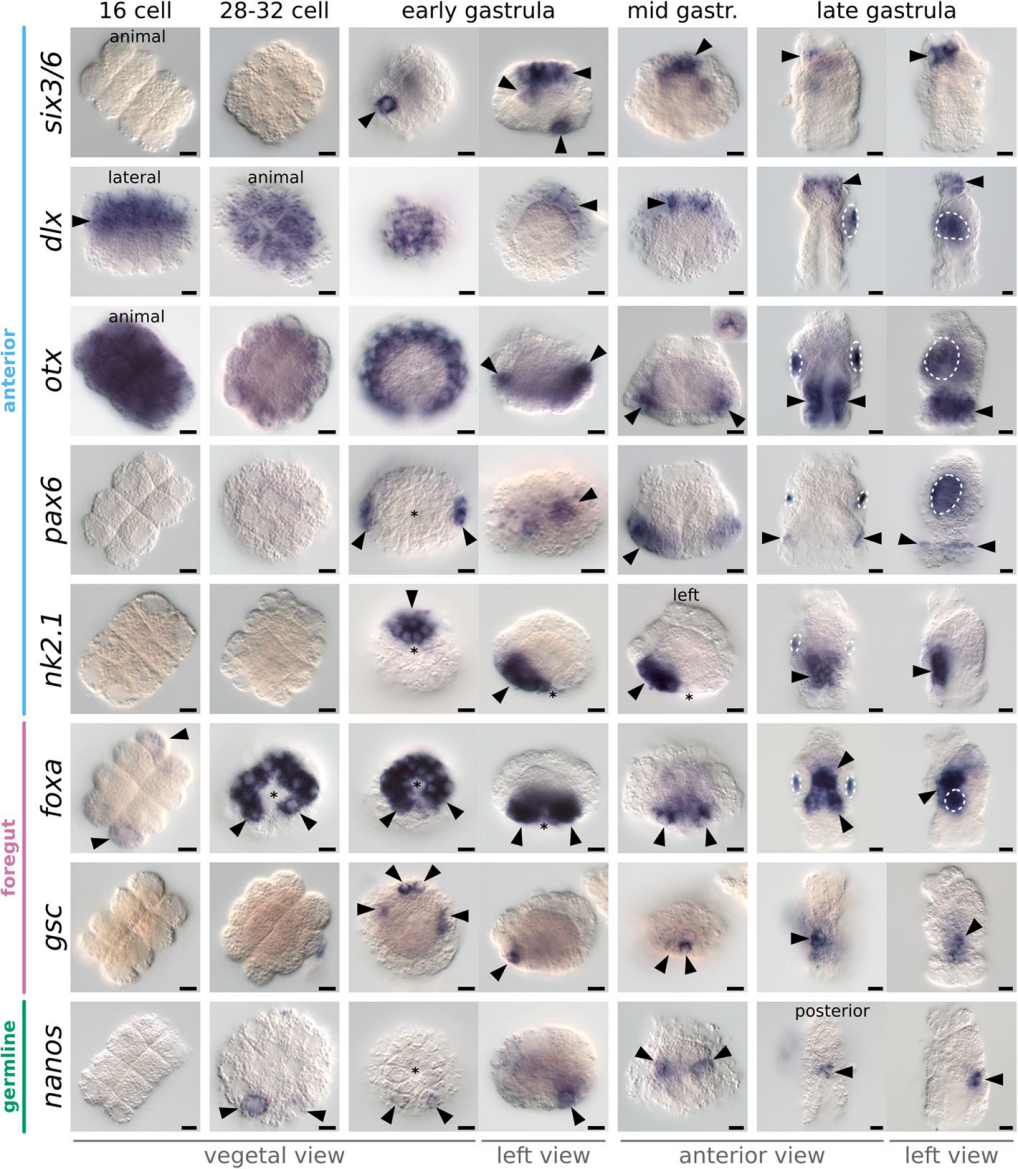
In situ hybridization of anterior, foregut, and germline markers during *M. membranacea* embryonic development. Orientation of the embryos is indicated below each column and exceptions are labeled on individual panels. In vegetal views, the B quadrant is *top* and D quadrant is *bottom*. In left views, the B quadrant (anterior region) is to the *left*. In all views, except for vegetal views, the animal pole is *top*. *Arrowheads* indicate expression and *dashed areas* mark unspecific staining attached to the shell valves of some embryos. *Asterisks* indicate the position of the blastopore. 16-cell = 5.2 hpa, 28- to 32-cell = 6.7–7.5 hpa, early gastrula = 11 hpa, mid gastrula = 16 hpa, and late gastrula = 24 hpa. Scale bar = 10 μm

**Fig. 10:**
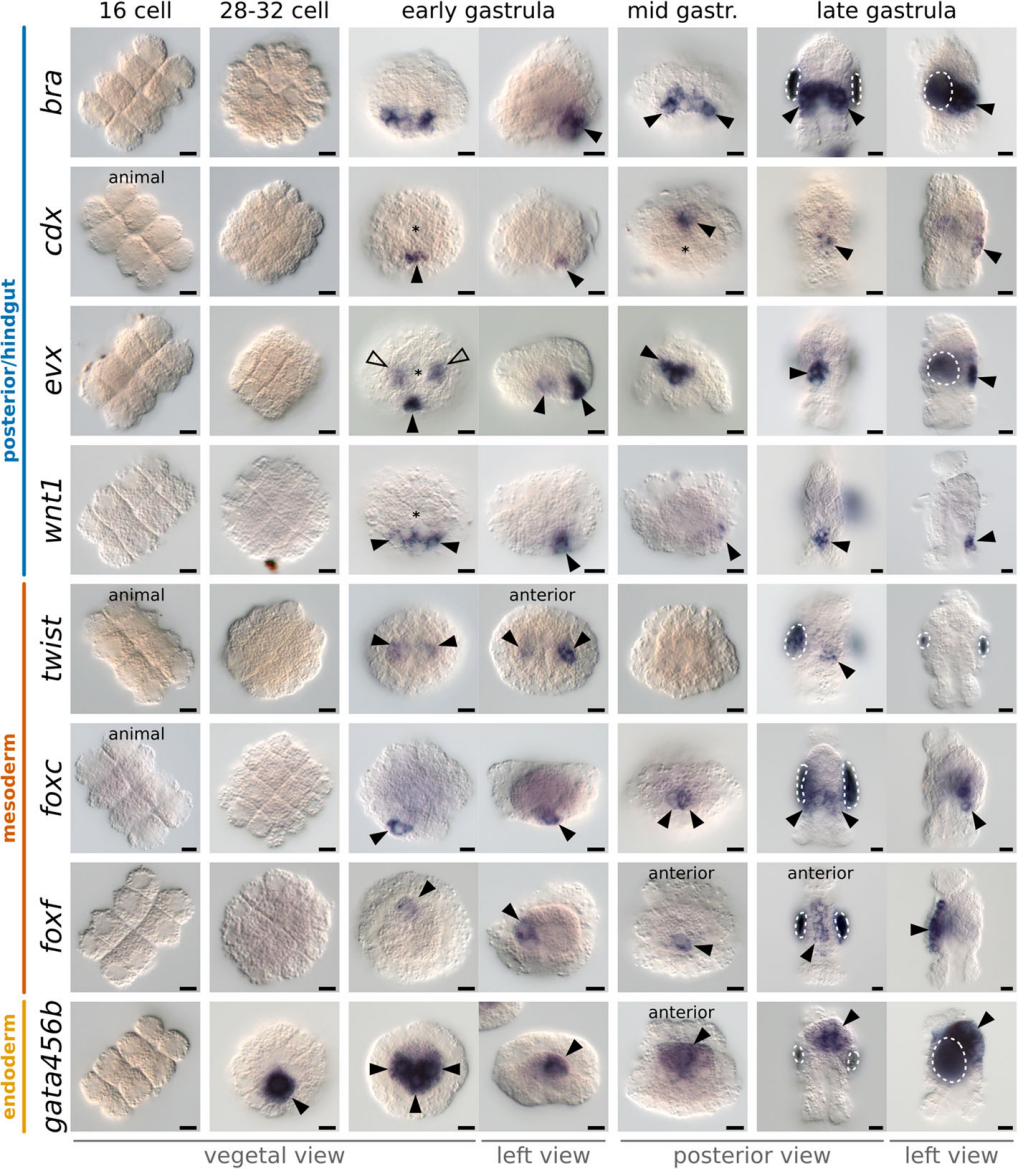
In situ hybridization of posterior/hindgut, mesoderm, and endoderm markers in the development of *M. membranacea*. Orientation of the embryos is indicated below each column and exceptions are labeled on individual panels. In vegetal views, the B quadrant is *top* and D quadrant is *bottom*. In left views, the B quadrant (anterior region) is to the *left*. In all views, except for vegetal views, the animal pole is *top*. *Arrowheads* indicate expression and *dashed areas* mark unspecific staining attached to the shell valves of some embryos. *Arrowhead outlines* indicate expression at a different focus plane. *Asterisks* indicate the position of the blastopore. 16-cell = 5.2 hpa, 28- to 32-cell = 6.7–7.5 hpa, early gastrula = 11 hpa, mid gastrula = 16 hpa and late gastrula = 24 hpa. Scale bar = 10 μm

**Fig. 11:**
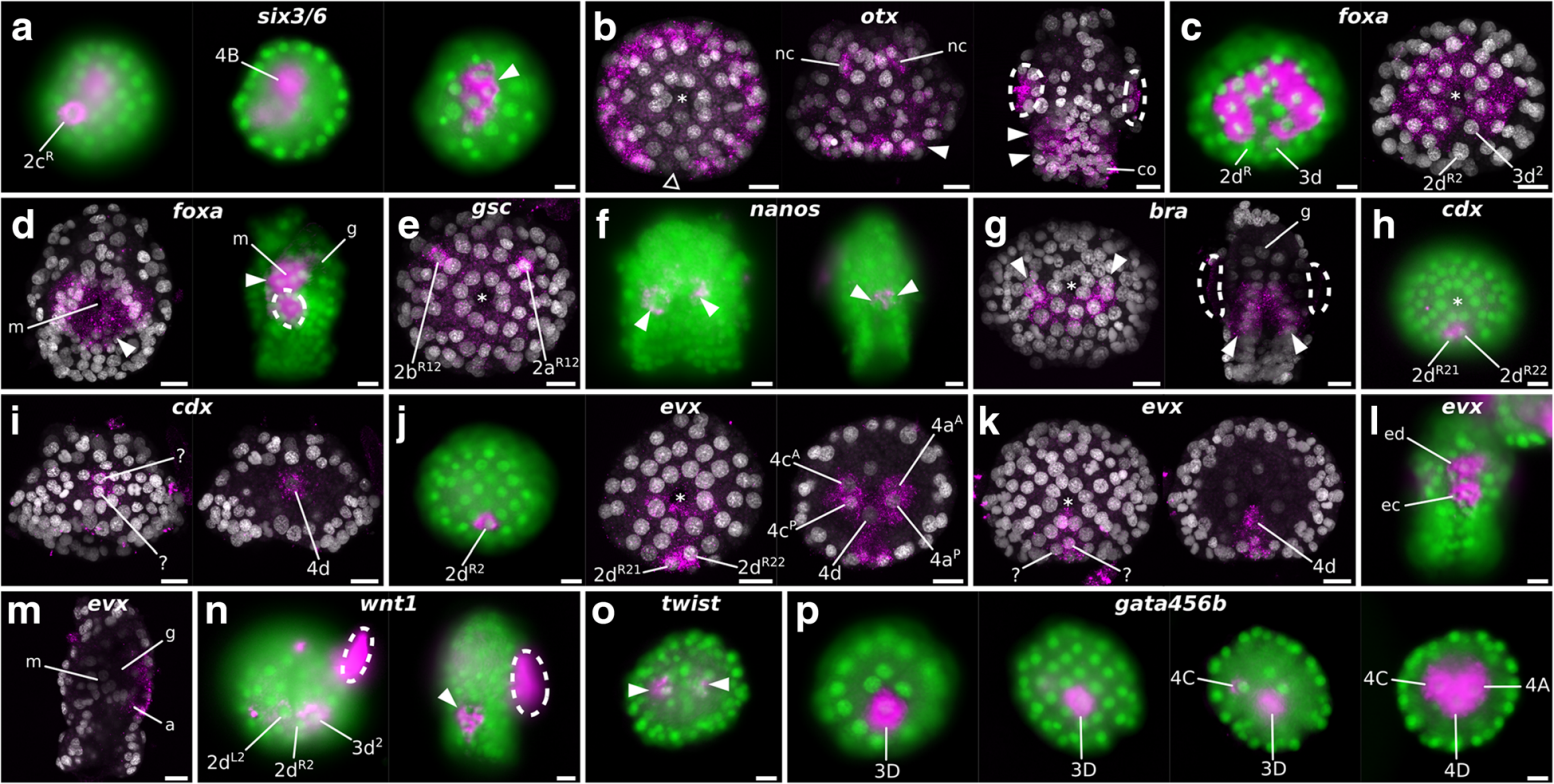
Gene expression details with cell resolution in *M. membranacea*. Selected embryos from the in situ hybridizations shown in Figs. 9 and 10 observed under a compound microscope with a fluorescent lamp (*green* = nuclei, *magenta* = signal) or maximum intensity projections from confocal microscopy (*gray* = nuclei, *magenta* = signal). *Arrowheads* point to the relevant areas of gene expression while *dashed areas* mark unspecific background staining. Asterisks mark the position of the blastopore. **a** Expression of *six3/6* at different focal levels. **b** Expression of *otx* in a vegetal view (*left*) showing the posterior gap in expression (*triangle outline*), neural cells at the apical disc (*nc*) of a mid gastrula embryo and the wider expression in the late gastrula (*arrowheads*). **c** Expression of *foxa* at 9.7 hpa in the 90-cell stage (*left*) without signal on the posterior cells 2d^R^ and 3d, and the same posterior gap one cell division cycle later (*right*). **d** Late gastrula stage (*left*) viewed from the posterior vegetal end to show the mouth opening with surrounding expression of *foxa* (B quadrant is *bottom*). On the right, a left side view with *foxa* expression in the mouth region. **e** Bilateral anterior cells expressing *gsc*. **f** Two *nanos*-positive cells during mid gastrula (*left*) and late gastrula (*right*). **g** Posterior and lateral cells on the vegetal ectoderm expressing *bra* (*left*) and a posterior view of a late gastrula depicting the domain in the posterior epithelium of the vestibule (*right*). **h** Vegetal view of early gastrula with the two vegetal cells with *cdx* expression (2d^R21^ and 2d^R22^). **i** *cdx* expression observed in two cells at the posterior ectoderm (*left*), and in the 4d cell (*right*) at mid gastrula with two. **j** Expression of *evx* in one posterior ectodermal cell (2d^R2^) on the vegetal side during early gastrulation (*left*). Progeny of 2d^R2^ expresses *evx* (*center*) as well as the derivatives of 4a and 4c and the 4d cell. **k** Mid gastrula stage with *evx* expression in at least two posterior ectodermal cells (*left*) and in the 4d (*right*). **l** Posterior view of a late gastrula with *evx* expressed in the posterior endoderm (ed) and ectoderm (*ec*). **m** Left side view of *evx* expression at the late gastrula with posterior endodermal and ectodermal domains. **n** Expression of *wnt1* during early gastrulation is restricted to three cells, 2d^L2^, 2d^R2^, and 3d^2^ (*left*) and a posterior cluster of cells at the late gastrula (right). **o** *twist* expression in internalized blastomeres. **p** Expression of *gata456b* from 32-cell stage until early gastrulation. Transcripts are restricted to the 3D until the internalization of vegetal blastomeres, when 4A and 4C initiate the expression of *gata456b*. Scale bar = 10 μm

In order to complement the cell lineage data, we cloned the bryozoan orthologs of 16 widely conserved patterning genes that have known and largely consistent developmental roles in diverse metazoans, namely the anterior markers *six3/6*, *dlx*, *otx*, *pax6*, and *nk2.1*; the foregut genes *foxa* and *gsc*; the germline marker *nanos*, the posterior and hindgut genes *bra*, *cdx*, *evx*, and *wnt1*; the endodermal marker *gata456*; and the mesodermal genes *twist*, *foxc*, and *foxf*. We analyzed the expression of these genes during *M. membranacea* development (Figs. 9, 10 and 11; Additional file 15: Figure S9), and used them as markers to further reveal the identities of the different blastomeres in the bryozoan embryo.

We first detected transcripts of *six3/6* – a transcription factor associated to anterior ectodermal patterning in cnidarians [61], hemichordates [62], and diverse protostomes [63] – during early *M. membranacea* gastrulation in one outer lateral vegetal plate cell (2c^R2^), one anterior endomesodermal cell (4B), and in five cells of the apical disc (Figs. 9 and 11a). Expression of *six3/6* clears from 2c^R2^ and 4B, but persists in the inner cells of the forming apical organ, a central neural region occupied by serotonergic-positive cells in other cyphonautes larvae [64]. We detected *dlx* transcripts, a gene involved in neurogenesis and proximodistal patterning of flies and vertebrates [65], in the eight animal pole cells (1q) of the 16-cell stage (5.2 hpa), broadly in the apical disc during gastrulation and elongation and, finally, localized to the whole apical organ in the late gastrula (Fig. 9).

The gene *otx* is involved in anterior ectodermal patterning of diverse metazoans [62, 63, 66–71] and endomesoderm specification of deuterostomes [72–74]. In *M. membranacea*, *otx* is expressed in all blastomeres between the 2- and 8-cell stages and gets restricted to the apical octet of the 16-cell stage at 5.2 hpa (Fig. 9). At the 32-cell stage (7.5 hpa), *otx* transcripts localize to the 1q^2^ octet and during gastrulation there are three rows of cells expressing *otx* with a posterior gap (Fig. 11b). During mid gastrula (16 hpa), two cells in the apical organ express transcripts of *otx* (Fig. 11b). In the late gastrula (24 hpa), *otx* is expressed in the corona and vestibule epithelium (Fig. 11b). Expression of *pax6* is first detected during gastrulation, in bilateral patches of the animal ectoderm, and remains as a thin line of expression encircling the embryo above the corona (Fig. 9). The gene *nk2.1* is involved in the patterning of the neural plate in vertebrates [75] and is expressed in anterior and ventral territories, including the apical/neural plate and anterior endoderm in cephalochordates [76], hemichordates [62], echinoderms [77], and annelids [71]. Transcripts of *nk2.1* are present in the progeny of the vegetal cells 2b and 3b in the early gastrula stage at 9.7 hpa (Fig. 9). These cells occupy an anterior vegetal position abutting the anterior blastopore lip until the edge of the vegetal plate. After the invagination of the vegetal plate, *nk2.1*-positive cells are lining the anterior portion of the preoral funnel, next to the mouth.

Expression of *foxa* is related to endoderm specification and commonly associated with the blastopore lip and foregut tissues in echinoderms [78] and annelids [79, 80]. At the 16-cell stage (5.2 hpa), we detected faint expression of *foxa* in the outer vegetal blastomeres and in 10 (out of 12) cells surrounding the four large blastomeres at the 32-cell stage (2q and 3q, except posterior cells 2d^L^ and 2d^R^) (Figs. 9 and 11c). Expression of *foxa* persisted in the daughter cells of the next division forming two rows of cells around the blastopore with a gap at the posterior end (Fig. 11c). With the invagination of the vegetal plate, this region occupies an anterior/lateral position in the vestibule wall, surrounding the mouth region of the late gastrula (Figs. 9 and 11d). We only found transcripts of *gsc* at the early gastrula stage (9.7 hpa) in two anterior and a bilateral pair of cells at the vegetal plate (Figs. 9 and 11e). In the late gastrula (24 hpa), *gsc* is expressed in bilateral domains of the vestibule wall, which fuse anteriorly.

The widely conserved germline marker *nanos* [81, 82] is expressed at 7.5 hpa in two posterior cells of the vegetal plate at the 32-cell stage (2d^L^ and 3d) (Fig. 9). In subsequent stages, *nanos* continues to be restricted to two cells at the posterior portion of the vegetal plate, localizing to the internal sac region of the cyphonautes larva (Fig. 11f).

The posterior/hindgut and mesodermal markers that we tested only initiate expression during gastrulation. The gene *bra* can have multiple roles, but it is generally related to mesoderm and posterior/hindgut patterning in several metazoans [83]. Expression of *M. membranacea bra* at 9.7 hpa in the early gastrula occurs at the vegetal plate in a posterior band of cells near the blastopore lip (Figs. 10 and 11g). It localizes to 6–8 cells at the posterior end of the mid gastrula and a broad portion of the posterior and lateral vestibule ectoderm (Fig. 11g). *M. membranacea bra* expression domain reaches the posterior portion of the preoral funnel as well as the future hindgut area of the larva (Fig. 10). A single posterior vegetal plate cell (2d^R2^) and its daughter cells (2d^R21^ and 2d^R22^) express the posterior/hindgut markers *cdx* and *evx* at the early gastrula (Figs. 10 and 11h, j). During gastrulation, *cdx* and *evx* continue to be expressed at the posterior edge of the vegetal plate (Figs. 10 and 11j, l) and localize to the posterior vestibule ectoderm (hindgut) of the late gastrula (Figs. 10 and 11l). At this stage, *evx* is also found in the posterior region of the gut (Fig. 11l, m). We also detected a transient *evx* expression in the two internalized blastomeres 4a and 4c of the early gastrula. Finally, *wnt1* is expressed in a row of 3–5 cells (including 2d^L2^, 2d^R2^, and 3d^2^) posterior to the blastopore during gastrulation (Figs. 10 and 11n). At the late gastrula (24 hpa), *wnt1* is detected at the posterior-most vestibule ectoderm, positioned between the corona and hindgut (Figs. 10 and 11n).

Expression of *twist*, a central regulator in mesoderm differentiation in several metazoans [84], occurs in a narrow time window in the early gastrula of *M. membranacea*. We detected a colorimetric signal in bilateral internalized cells of the early gastrula – possibly 4a, 4c, or derivatives – as well as at the anterior end of the late gastrula (Figs. 10 and 11o). Transcripts of *foxc*, commonly expressed in anterior and posterior mesodermal domains in flies [85], annelids [86], and brachiopods [87], are present in one unidentified posterior vegetal plate cell of the early gastrula and two similarly positioned cells during mid gastrulation (Fig. 10). In the late gastrula, *foxc* expression is located in the internal sac area. The gene *foxf* is a transcription factor involved in mesoderm patterning and expressed mainly in visceral and anterior territories in flies [88, 89], cephalochordates [86, 90], and brachiopods [87]. In *M. membranacea* it is expressed in the mesodermal cell 4b in the early and mid gastrula stages (Fig. 10). This cell and its descendants divide subsequently from basal to apical, forming a distinct frontal row of mesodermal cells expressing *foxf* at the anterior portion of the late gastrula.

We found two copies of the endomesodermal marker *gata456* [91] in the transcriptome of *M. membranacea*. While the gene *gata456a* is not expressed at detectable levels in any of the analyzed stages, *gata456b* is strongly expressed in endodermal tissues of the bryozoan. The expression of *gata456b* initiates early, in the vegetal 3D blastomere at the 32-cell stage (7.5 hpa) (Figs. 10 and 11p). The expression expands to adjacent lateral blastomeres 4A and 4C in the early gastrula, and in subsequent stages *gata456b* continues to be expressed in the endodermal tissues forming the gut of the cyphonautes larva (Figs. 10 and 11p).

## Discussion

The phylogenetic position of bryozoans provides a valuable opportunity to investigate the evolution of developmental traits. Even though the kinship of Bryozoa remains inconclusive – the group is more closely related either to Entoprocta and Cycliophora [92] or to Phoronida and Brachiopoda [29], or both [32] – most phylogenetic analyses place the bryozoans nested within the Spiralia [27, 29–32]. This indicates that the ancestral cleavage pattern of Spiralia – spiral cleavage – must have been modified in the bryozoan lineage during evolution [5]. Here, we examined the similarities and differences between the embryogeneses of the bryozoan *M. membranacea* and those of spiral-cleaving embryos, by integrating cell lineage and molecular data, and provide a hypothesis for the evolution of bryozoan development from a spiral-cleaving ancestor.

### Specification of the D quadrant

One critical event of animal embryogenesis is the establishment of dorsoventral polarity. In spiral-cleaving embryos, this event is tied to the specification of the D quadrant during development [93]. In species where the first two embryonic cell divisions are unequal, the D quadrant is determined early by the asymmetric distribution of maternal cytoplasmic determinants, while in spiral-cleaving species that form equal-sized blastomeres at the 4-cell stage, the D quadrant is specified around the 24- to 32-cell stage by inductive interactions mediated by cell contacts between micromeres and macromeres [93–97]. In the current work, we found evidence that the specification of the D quadrant in the equal, biradial-cleaving bryozoan *M. membranacea* resembles that of equal, spiral-cleaving molluscs in the timing of specification, pattern of MAPK activation, and asynchrony of the D quadrant cell divisions post-specification.

In equal-cleaving molluscs, the specification of the D quadrant correlates with the activation of the MAPK pathway in the 3D macromere only [55, 57]. In *M. membranacea*, whose equal-sized blastomeres at the 4-cell stage give rise to perfectly symmetrical embryonic quadrants, that are indistinguishable from each other until gastrulation, the earliest molecular asymmetry we could detect is the activation of the MAPK pathway in a single vegetal blastomere that produces the posterior portion of the larval body. As in equal-cleaving molluscs, MAPK is activated in the bryozoan 3D blastomere on the fifth round of cell divisions, suggesting the D quadrant of *M. membranacea* is specified as early as the 28-cell stage. This might indicate that bryozoans and equal-cleaving molluscs undergo similar developmental mechanisms of D quadrant specification (but see below). Interestingly, most equal-cleaving spiralians studied so far exhibit a single MAPK-activated blastomere during early development, while unequal-cleaving species show diverse patterns of activation (see Additional file 16: Table S1), thus suggesting that this pattern of MAPK activity is a common feature of equal-cleaving embryogenesis independent of its cleavage geometry.

Blocking the MAPK pathway during mollusc embryogenesis results in radialized larvae that lack muscles, shell, and foot, suggesting that MAPK activation might signal for the specification of the D quadrant [54–57]. We tested if the MAPK pathway could have a similar developmental role in the bryozoan by using the MEK inhibitor U0126 [98]. We found that blocking the MAPK pathway early in development severely disrupts the normal development of *M. membranacea*, a phenotype analogous to the radialized larvae of molluscs. However, later treatments do not result in axial defects, even if the drug is applied before the activation of the MAPK in the 3D blastomere. Thus, the occurrence of the severe phenotype does not correlate with the period of MAPK activation in *M. membranacea*, in contrast to the snail *Crepidula fornicata*, where the embryos become radialized in all treatments before and during MAPK activation – but are not disrupted if the drug is applied after this critical period [56].

The fact that blocking the 3D MAPK activation does not lead to axial defects in the bryozoan suggests that the MAPK pathway might not have a role in specifying the D quadrant in *M. membranacea*. However, our experimental dataset does not exclude alternative explanations. One possibility is that the U0126 concentration that we used for the timed experiments (10 μM) is not sufficient to completely inactivate MEK, and the remaining MAPK (ERK) – although undetected by immunohistochemistry – would still transduce the signal and form larvae without axial defects. For instance, similar mildly abnormal phenotypes were also observed in molluscs treated with the same U0126 concentration, which can indicate the partial inactivation of the MAPK signaling [57, 99]. In addition, we cannot exclude the possibility that the early disruption we observe in *M. membranacea* is due to an undetected period of MAPK activation before the 3D activation, or even due to off-target effects of the inhibitor. For these reasons, although we present preliminary evidence that MAPK inhibition alters *M. membranacea* development, the developmental role of the MAPK pathway in bryozoans remains unclear and dependent upon future work using higher U0126 concentrations and additional experimental conditions.

Once the D quadrant has been determined, it typically shows asynchronous cell divisions in relation to the other quadrants of spiral-cleaving embryos [100]. For instance, the 3D macromere in the mollusc *Patella vulgata* [94] and the 1d derivatives of *Ilyanassa obsoleta* [101, 102] undergo a late division. Our analyses of *M. membranacea* cell lineage indicate similar asynchronous cell divisions in the D quadrant, which include the 3D blastomere and 1d derivatives of the bryozoan. Therefore, the specification of the D quadrant seems to be correlated with subsequent changes in the cell cycle timing in both *M. membranacea* and spiral-cleaving embryos.

Overall, *M. membranacea* exhibits a similar pattern and timing of MAPK activation, as well as equivalent asynchronous cell divisions in the D quadrant, when compared to equal-cleaving molluscs. Given the phylogenetic position of bryozoans, these similarities might suggest that some of the underlying traits of spiral-cleaving embryos were maintained during the evolutionary transition from spiral to biradial cleavage. The comparison also reveals that equal cleavage might be associated with a single D quadrant MAPK-activated blastomere in spiralian development. Nevertheless, the MAPK pathway is still poorly sampled in spiralians, and other spiral and non-spiral-cleaving groups, such as phoronids, nemerteans, polyclads, rotifers, and gastrotrichs, need to be investigated to properly understand the roles and the evolution of MAPK signaling in spiralian development.

### Comparative spiralian fate maps

The stereotypic nature of spiral cleavage supports the identification of putative homologous blastomeres between different spiralian lineages, and therefore enables the unprecedented comparison of blastomere fates across clades [6, 9, 12, 103, 104]. The comparative study of spiral cleavage has revealed that homologous blastomeres share mostly-similar fates in various clades [5, 18, 19, 48, 105]. The cleavage of *M. membranacea* clearly differs from the spiral cleavage pattern, which complicates the identification of homologous blastomeres between the bryozoan and a spiral-cleaving embryo. However, we established a common developmental feature to base our comparative cell lineage and gene expression analyses.

**Fig. 12:**
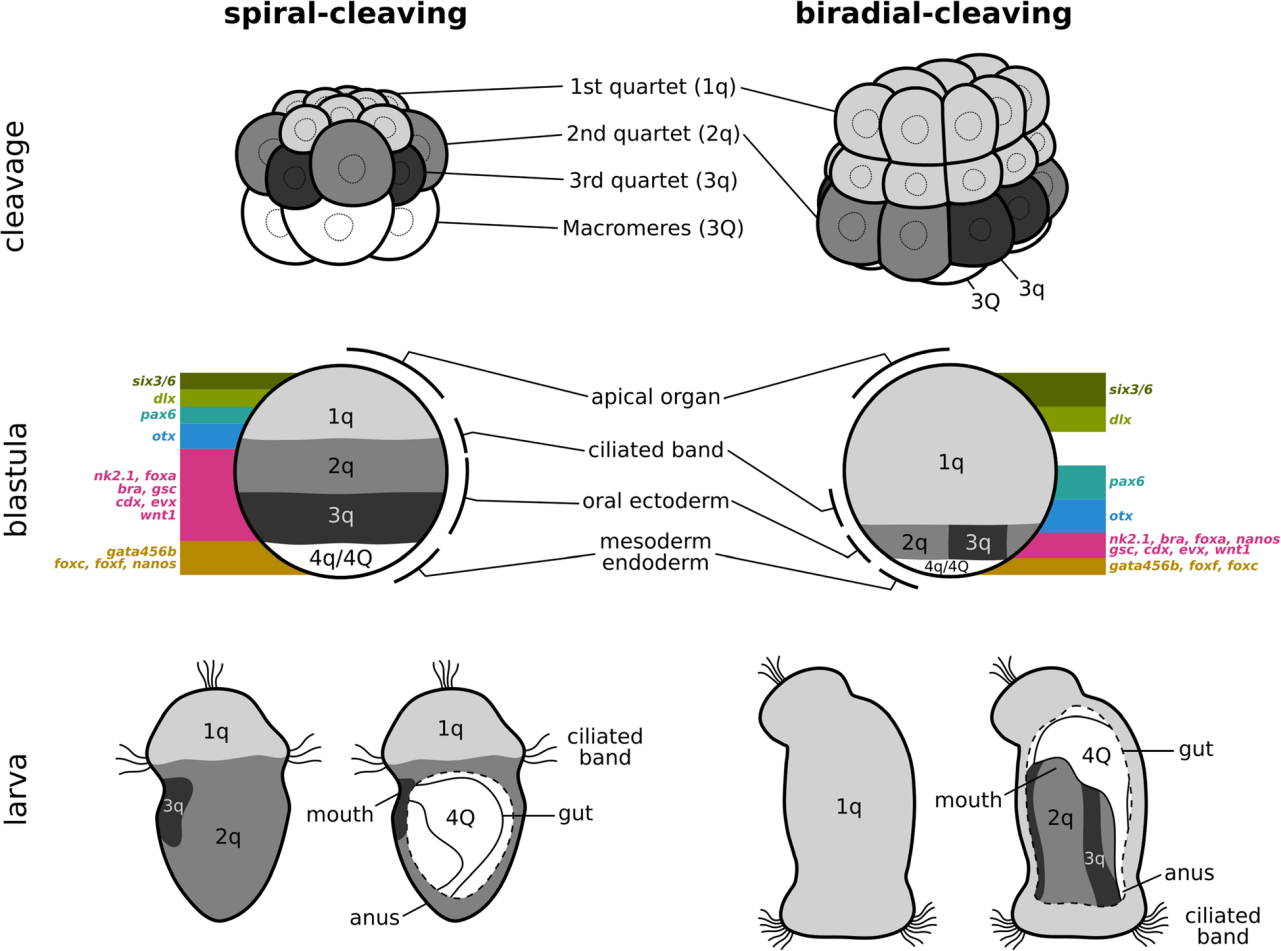
Bryozoan development in comparison to spiral cleavage. Line drawings represent cleavage, blastula, and larval stages of a generalized spiral-cleaving embryo (based on [19]) and the biradial-cleaving embryo of the bryozoan *M. membranacea*. Shades of grey indicate the first, second, and third quartets and their respective fates in the blastula and larval tissues. The fourth quartet, macromeres, and descendants are depicted in white. A simplified summary of the gene expression domains is mapped to the blastula stage

In both spiral and bryozoan embryogenesis, the vegetal blastomeres sequentially give rise to quartets of daughter cells, while remaining at the vegetal-most portion of the embryo until being internalized during gastrulation. We thus compare the quartets of *M. membranacea* to the quartets of spiral-cleaving embryos in terms of gene expression and fate in the larval tissues. We find the quartets have a similar molecular identity and contribute to the same set of structures in the larvae of bryozoan and spiral-cleaving groups, and that the subset of blastomeres that gives rise to these structures partially overlaps (Fig. 12). This indicates that bryozoans might share a common embryonic patterning of early blastomere fates with other spiralians, and that, in the current phylogenetic scenario, such developmental trait has remained conserved despite the drastic modification in the cleavage pattern from spiral to biradial.

#### First quartet: apical organ and ciliated band

The first quartet of micromeres in spiral-cleaving embryos contributes to the apical organ, the ciliated band, and all the ectoderm in between [6, 9, 11, 106–114]. In the *M. membranacea*, the first quartet of animal blastomeres also gives rise to these ectodermal structures of the cyphonautes larva. This suggests that, in both the bryozoan and spiral-cleaving embryos, the third cleavage demarcates a split in the embryonic fate map, in which the first quartet of animal blastomeres only gives rise to the ectodermal structures placed towards the animal pole, while the progeny of the vegetal blastomeres (i.e., the second, third, and fourth quartets) produces a different set of larval structures (see the next sections). When we compare in more detail the specific fates of the descendants of the first quartet, we find that some blastomeres contributing to the apical organ or ciliated band of the cyphonautes larva indeed contribute to the respective structures of spiral-cleaving larvae – but that this similarity is not complete, and different subsets of blastomeres contribute to the apical organ and ciliated band.

The apical organ, for example, is usually formed by the progeny of the apical-most 1q^1^ micromeres in groups with spiral cleavage [48, 105]. While the apical organ of *M. membranacea* larva is also derived from the apical-most subset of 1q_i_^1^ (=1q^11^), descendants of 1q_e_^1^ (=1q^21^) also contribute to the structure. We find a similar situation when comparing the embryonic origin of the corona (i.e., the ciliated band of the cyphonautes larva) with the prototroch – a ciliated band considered to be an ancestral trait for the larval stages of trochozoan spiralians [115]. The prototroch of annelids and molluscs is formed by 1q (accessory and primary trochoblasts) and 2a–c (secondary trochoblasts) descendants [106, 113, 116]. Our data reveals that the corona of *M. membranacea* is formed by blastomeres equivalent to the accessory and primary trochoblasts of the prototroch [106], but unlike spiral-cleaving embryos, the second quartet does not contribute to the ciliated band of the bryozoan larva (Additional file 17: Figure S10). Thus, despite being located in a vegetal domain of the bryozoan embryo, such as the telotroch of some spiral-cleaving larvae, the corona shares a common embryonic origin with the prototroch.

In general, we find that equivalent early blastomeres of the bryozoan and spiral-cleaving embryos contribute to similar larval structures, but that the fate of the progeny of these early blastomeres only partially overlap between the bryozoan and spiral-cleaving embryos. These observations suggest that, during the evolution of the bryozoans, shifts in the blastomere fates occurred in late embryogenesis while the early embryonic patterning, presumably inherited from a spiral cleavage ancestor, might have remained conserved.

The bryozoan fate map indicates that the apical organ, outer ectoderm, and corona of the cyphonautes larva have a similar embryonic origin as the apical organ, pretrochal elements, and prototroch of spiral-cleaving embryos, respectively. They all derive from the first quartet blastomeres. The fate map similarity is paralleled by molecular data, since the genes expressed in this region of *M. membranacea* have an equivalent spatial arrangement in other spiralian embryos. *six3/6* and *dlx* are expressed at the animal end, while *otx* is expressed in the vegetal-most progeny of the first quartet. Other spiralian embryos display a similar arrangement of these transcripts [63, 68, 69, 71, 117–122] (Additional file 18: Table S2). Therefore, the region derived from the first quartet of *M. membranacea* match the pretroch region of spiral-cleaving embryos.

In *M. membranacea* the blastomeres that form the apical organ express *six3/6* and *dlx* from the 16-cell stage, suggesting that these genes might be involved in the establishment of the embryonic animal–vegetal identities, and possibly in the molecular patterning of the cyphonautes apical organ. The expression of *otx* in the bryozoan is associated with the ciliated band of the cyphonautes larva, similar to other spiralians where *otx* is expressed near or in the larval ciliated band [68, 69, 123]. The gene is an interesting example because it provides the opportunity to integrate cell lineage and gene expression data between the bryozoan and spiral-cleaving embryos. As explained above, the ciliated band of trochophore larvae, the prototroch, is formed by the contribution of first quartet and second quartet blastomeres [106, 116], while the corona of *M. membranacea* derives solely from first quartet blastomeres, which are putatively homologous to the primary trochoblasts of the prototroch. In the mollusc *Patella vulgata*, *otx* is expressed in all prototroch cells [123]. Interestingly, the second quartet blastomeres of *M. membranacea* – the set of blastomeres that form the secondary trochoblasts in the prototroch – also express *otx*, as observed in *P. vulgata*, even though these cells do not contribute to the corona of the cyphonautes larva. This observation suggests that presumptive homologous blastomeres between the bryozoan and other spiralians might still share a similar molecular identity, even though they do not form similar tissues.

Overall, our work reveals that the first quartet of *M. membranacea* embryo and the first quartet of spiral-cleaving embryos give rise to a similar set of larval structures, and give rise to a larval body region with similar molecular profile. Thus, the outer ectodermal region of the cyphonautes larva corresponds, in developmental terms, to the head region of other spiralians.

#### Second and third quartet: larval mouth

The second and third blastomere quartets of spiral-cleaving embryos contribute to a diverse set of ectodermal structures, such as the foregut, ciliated bands, neurons, the mollusc shell gland and foot, the annelid trunk and nerve cords, as well as ecto-mesodermal muscle cells [107–114, 124, 125]. In *M. membranacea*, these blastomeres form the whole vegetal ectoderm that gives rise to the vestibule epithelium, including the preoral funnel and posterior ectoderm of the cyphonautes larva. In most spiralians, the second and third quartets are the blastomeres surrounding the blastopore – the orifice formed at the site of endomesoderm internalization [126], whose developmental fate has been a significant trait for the discussions about metazoan evolution [127–129]. Nevertheless, the fate of the blastopore in bryozoans remained open to discussion [39, 41, 44, 49].

Even though in most gymnolaemate bryozoans the blastopore closes after gastrulation [41, 43], or in some cases, an orifice is not formed at all [42], an ultrastructural study in *M. membranacea* revealed that its blastopore remains open until the larval stage [49]. Our cell lineage data indicate that cells at the blastopore lip give rise to the preoral funnel of *M. membranacea*, and that the endodermal cells lining the blastopore form the anterior portion of the larval gut. We also found the foregut marker *foxa* is expressed around most of the blastopore lip, except for a couple of posterior rows, and that *foxa* expression persists around the future larval mouth, indicating that most cells associated with the blastopore of *M. membranacea* have a foregut molecular identity. Thus, independent ultrastructural, molecular, and cell lineage data provides robust evidence for a persistent blastopore and the protostomic development of *M. membranacea*, as previously suggested [49].

We found that the vegetal ectoderm – the cells derived from the second and third quartet – exhibits an anteroposterior polarity, as revealed by the differential expression of molecular markers. The anterior/foregut markers *nk2.1*, *foxa*, and *gsc* are expressed in a region opposed to posterior/hindgut markers *bra*, *cdx*, *evx*, and *wnt1*, which are generally restricted to the D quadrant. Transcripts of *nk2.1* are restricted to the B quadrant in a comparable position, in relation to the cyphonautes anteroposterior axis, to the anterior/ventral expression found in other bilaterians [62, 71, 122, 129, 130]. In a similar fashion, transcripts of *wnt1*, a gene commonly expressed at the posterior end of bilaterians [131–134], occur at the posterior region of the vegetal ectoderm of *M. membranacea* (see also Additional file 18: Table S2 for a comparison of spiralian-specific gene expression patterns). This suggests that at least some molecular aspects of the bilaterian axial patterning has remained conserved in the cyphonautes larva.

In some cases, the transcripts of *M. membranacea* are not only located at similar body regions, but in the putative homologous blastomeres of spiral-cleaving embryos. An example is the expression of *foxa* between the bryozoan and the annelid *Hydroides elegans* [79]. In both, *foxa* is expressed in the second quartet blastomeres early in development and in the cells that surround the blastopore during gastrulation, with a peculiar posterior gap [79]. Another comparable cellular expression is the gene *bra*, expressed in the second and third quartet progeny at the posterior lip of the blastopore of the molluscs *Patella vulgata* [99] and *Haliotis asinina* [57]. Therefore, the *M. membranacea* data indicates that the molecular identity of the blastomeres remained conserved to a certain extent within spiralians, despite the modified cleavage geometry and vegetal placement of the second and third quartet in the bryozoan embryo.

#### Fourth quartet: muscle and mesenchymal cells

The embryonic source of mesoderm in bryozoans has been a contentious topic [49]. Classical works suggest that mesodermal cells derive from endodermal blastomeres, but could not demonstrate the embryonic origin with cellular resolution [40–43, 45, 135]. However, recent ultrastructural data in *M. membranacea* suggests an ectodermal origin for the bryozoan mesoderm, from the delamination of an ectodermal cell during gastrulation [49]. Our cell lineage data indicate that the first mesodermal cells of *M. membranacea* derive from the fourth quartet (4a–4c). The lateral cells 4a^A^ and 4c^A^ form the anterior muscles of the cyphonautes larva, while the progeny of 4b^1^ gives rise to a stack of mesenchymal cells that express the anterior mesoderm marker *foxf*. We did not observe the delamination of an anterior ectodermal cell as described by Gruhl [49], but cannot discard the existence of other cells contributing to the mesoderm of *M. membranacea*. Our work corroborates previous classical studies of bryozoan embryology by revealing that the mesoderm of the bryozoan *M. membranacea* originates from multiple fourth-quartet blastomeres.

The source of mesodermal tissues in spiral-cleaving embryos has been extensively studied and discussed [5, 9, 60, 108, 109, 112–114, 124, 125, 136–141]. There are generally two sources, an anterior mesoderm derived from the third quartet blastomeres (known as ectomesoderm) and a posterior mesoderm derived from the 4d blastomere. While the blastomeres contributing to the anterior mesoderm (usually 3a and 3b) are more variable [113, 141], most spiral-cleaving species have the 4d as the sole endomesodermal contributor. However, there are exceptions. In the annelid *Capitella teleta*, for example, mesoderm formation shifted to the 3c and 3d blastomeres [10, 114], even though the 4d blastomere still produces the germline [142]. This reveals how a particular contribution to later tissues can be decoupled and geared to different blastomeres in spiral-cleaving embryos [113]. We find that the source of mesodermal tissues of the bryozoan *M. membranacea* differs from other spiralians because (1) the third quartet does not contribute to the anterior mesoderm and (2) multiple blastomeres of the fourth quartet give rise to mesodermal tissues. In addition, the blastomeres 4a–4c often give rise to endodermal tissues in spiral-cleaving embryos [108, 113]. Therefore, our data suggests that the specification of the anterior mesoderm in the bryozoan might have been shifted in time and in position from the third to the fourth quartet.

Although only a subset of the genes we analyzed is expressed in the mesoderm of *M. membranacea* (*six3/6*, *evx*, *twist*, *foxf*), the patterns indicate that the bryozoan mesoderm is already regionalized at early gastrulation. For instance, we found that 4d expresses *evx* and *cdx*, genes commonly associated to posterior mesodermal and hindgut fates in spiralians [120, 121, 129, 143–149]. Even so, we could not resolve the fate of the 4d blastomere in *M. membranacea* although the expression data indicates that the 4d blastomere might contribute to the posterior mesoderm and hindgut of the bryozoan. The lateral mesodermal cells 4a/4c and derivatives express *evx*, and possibly *twist*, in the same manner as the expression of *evx* orthologs in the annelid *Capitella teleta* during early development [150]. Finally, at the anterior mesoderm, we find the expression of *foxf*, also observed in the brachiopod *Terebratalia transversa* [87]. Overall, these molecular data reveal that *M. membranacea* mesoderm is regionalized and that at least some of the expression patterns are conserved with other spiralians.

The 4d cell and its descendants also form the germline and are known to express *nanos* in spiral-cleaving embryos [151]. Germ cells have not been identified during the embryogenesis of any bryozoan and were only found in zooids after metamorphosis [38]. The expression of *nanos* in *M. membranacea* differs from the pattern of spiral-cleaving embryos, since *nanos* is expressed in two posterior cells of the second and third quartet. These blastomeres divide repeatedly, but *nanos* expression is always retained in two cells that become part of the larval internal sac – the structure that persists during metamorphosis giving rise to the outer case of the zooid [152]. Thus, we hypothesize the *nanos*-positive cells might be stem cells contributing to the differentiation of the internal sac, but further analysis in competent larvae and metamorphosed juveniles are needed to clarify the fate and molecular identity of these cells.

#### 4Q blastomeres: gut

The 4Q blastomeres are the largest and yolkier cells of the gastrulating bryozoan embryo and, in *M. membranacea*, they produce the cyphonautes larval gut. Similarly, the macromeres of spiral-cleaving embryos generally give rise to endodermal tissues [6, 9, 11, 103, 108, 110–114, 153], even though in the polyclad *Hoploplana inquilina* these cells break up and the gut is likely derived from 4d [16, 109]. Nevertheless, the endodermal fate of these large vegetal cells appears to be a common feature between *M. membranacea* and spiral-cleaving embryos.

The expression of the endodermal marker *gata456b* corroborates the molecular identity of the 4Q blastomeres in the bryozoan. In spiralians, *gata456* expression is mainly associated to endodermal and, in some cases, mesodermal tissues [80, 87, 121, 129, 154–156]. The expression of *gata456b* in *M. membranacea* is clearly associated with the larval gut, suggesting that the molecular patterning of endodermal structures in the bryozoan is conserved with other spiralian groups, and possibly to other bilaterians [157]. One exception is the bryozoan *Bugula neritina*, where *gata456* is expressed at the apical organ [158]. However, this might be related to the fact that the coronate larva of *B. neritina* does not have a gut, and requires further investigation. The early expression of *gata456b* in *M. membranacea* does differ slightly from other spiralians, since neither the 4B blastomere nor the 4a–4c blastomeres show *gata456* transcripts [80, 154, 156]. Nevertheless, the common fate and *gata456* expression between *M. membranacea* 4Q blastomeres (4A, 4C, and 4D) and the macromeres of other spiralians suggest that the bryozoan shares similar molecular and developmental traits for the endoderm patterning with spiral-cleaving embryos.

### A modified spiral cleavage

Our investigation shows a series of similarities between the embryonic development of the bryozoan *M. membranacea* and the embryogenesis of annelids, molluscs, nemerteans, and polyclads. The vegetal blastomeres sequentially give rise to quartets of daughter cells, the first asynchronous cell divisions occur in the posterior quadrant, the quadrant identities can be identified at the 32-cell stage, the MAPK activity resembles that of equal-cleaving molluscs, and several genes are expressed in equivalent blastomeres or embryonic regions (Fig. 12). In addition, the early blastomeres of *M. membranacea* and of spiral-cleaving embryos have similar fates in the larval tissues (Fig. 12). That is, the first animal blastomeres form the whole region from the apical organ to the corona – equivalent to the pretrochal region – and the ciliated band itself, the second and third quartets contribute to the oral ectoderm, the fourth quartet gives rise to the mesoderm of the larva, and the four large vegetal blastomeres are internalized and become endoderm (Fig. 12). Since the phylogeny of Spiralia indicates spiral cleavage is ancestral and bryozoans are nested within the clade [27, 29–32], we interpret these developmental similarities as inherited traits from an ancestral spiral-cleaving embryogenesis.

In this context, during the evolution of gymnolaemate bryozoans the ancestral spiral cleavage pattern, characterized by the alternating oblique cell divisions, was modified to biradial cell divisions. While the cleavage pattern changed and the anterior mesoderm was reallocated to the fourth quartet, some aspects of the development have remained conserved, such as the D quadrant specification, MAPK activity, and overall fate map of early blastomeres.

Spiral cleavage has been modified not only in bryozoans, but in different spiralian branches as well, such as flatworms, molluscs, and brachiopods [5]. In most of these cases, the embryonic development has changed to such extent that no traces of spiral cleavage are found, and the ancestral cleavage geometry can only be inferred by the phylogenetic position of the clade (e.g., the discoidal cleavage of cephalopods [25]). Remnants of spiral cleavage usually consist of oblique mitotic spindles, as in the flatworm *Macrostomum lignano*, which displays a typical spiral cleavage pattern until the third cleavage, when the embryonic development becomes considerably modified [159]. The bryozoan *M. membranacea* differs from these previously known cases because we can recognize shared cell lineage and developmental traits with spiral-cleaving embryos that are not the cleavage geometry itself. The evolutionary mechanisms involved in the transition from spiral to biradial cleavage remain unclear, but changes in the orientation of the mitotic spindle have a genetic basis and are beginning to be uncovered using molecular and computational approaches [160–162]. Our data suggests that the quartet-divisions, cell fates, and other traits commonly associated with a spiral cleavage program were maintained in the bryozoan development despite the evolutionary modification to a biradial cleavage pattern.

### Evolution of cleavage patterns

The fate of the early embryonic blastomeres is thought to be causally related to the cleavage pattern during development [2, 163]. In such case, a change in the cleavage geometry would lead to a change in the cell fates. However, the bryozoan cell lineage illustrates a case where the cleavage pattern and blastomere fates are not evolutionary coupled. We find that the fates of the early blastomeres are similar between *M. membranacea* and spiral-cleaving embryos despite the modified biradial cleavage pattern. The relative positioning of the second and third quartets even differs between the bryozoan and a typical spiral-cleaving embryo (Fig. 12), but these blastomeres still contribute to similar tissues, suggesting the early cell fate determination remained relatively conserved during bryozoan evolution. The bryozoan cell lineage illustrates how a widely conserved determinate cleavage pattern – spiral cleavage – can evolve without major changes in other developmental traits, such as the blastomere fates and molecular identity.

We found several developmental genes expressed in a similar spatial arrangement between bryozoans and other spiralians, as revealed by MAPK (3D blastomere), *otx* (vegetal-most blastomeres of the first quartet), and *bra* and *foxa* (second and third quartets). This molecular map is similar not only to the typical spiral-cleaving embryos, but also to brachiopod embryos [87, 129], whose embryos have a much greater number of cells and no stereotypic cleavage pattern [164]. Thus, a single cell in the bryozoan embryo expressing *gata456* might be homologous to a whole region of *gata456* expression in the brachiopod embryo [87], as suggested by Hejnol [5]. This reinforces the hypothesis that cell fate determination is not tied to a particular cleavage pattern, but depends on the underlying molecular framework established early in development [165]. This is the case for nematodes, whose cleavage patterns diverged drastically between groups without a corresponding change in the resulting phenotype [4]. However, a clearer parallel case to the spiral-to-biradial evolution is the transition from the spiral cleavage of polychaete annelids to the derived cleavage of clitellate annelids; despite the differences in the cleavage pattern, the clitellate fate map does not deviate significantly from the annelid ground plan [166]. Overall, our findings support the hypothesis that, in evolutionary terms, the causal ontogenetic connection between cleavage pattern and blastomere fates, if any, can be broken [24]. In the case of the bryozoan *M. membranacea*, the molecular identity and fate of the early blastomeres might have been maintained, despite the modification in the geometry of cell divisions. Further comparative cell lineage studies with other non-spiral spiralian lineages, such as gastrotrichs and rotifers, will be crucial to establish the ancestral traits of spiralian development and to better comprehend the relation between cleavage patterns and cell fates during evolution.

## Conclusions

The embryonic development of *M. membranacea* provides a unique comparative standpoint to the typical spiral cleavage pattern. It reveals that spiral cleavage is not an all-or-nothing character and has been extensively modified in the diverse spiralian lineages. In particular, we suggest that the cleavage geometry of the bryozoan embryo evolved independently from other spiralian developmental traits. Therefore, modifying spiral cleavage does not require drastic developmental changes such as the ones found in cephalopods or parasitic flatworms. More generally, our data suggests that determinate cleavage patterns can be modified without major changes to the molecular identity of blastomeres and cell fates, which challenges the idea that the cleavage pattern is evolutionarily coupled to the specification of cell fates. In this perspective, the evolutionary conservation of cell fates in spiral-cleaving clades might be a consequence of a conserved underlying molecular patterning, overlaid by a determinate cleavage pattern. Overall, our work highlights the importance of comparative data to better understand the evolution of spiralian development.

## Methods

### Collection, spawning, and cultures

We collected *M. membranacea* in the fjord waters of Hjellestadosen (60°15′23.9" N 5°14′20.1" E) in Bergen, Norway, between May and September. We handpicked kelp blades with ripe bryozoan colonies from floating boat docks, and maintained the fronds in tanks with flowing sea water at 10 °C. To induce spawning, we cut a portion of the kelp blade with mature colonies, usually the ones with more opaque whitish/pinkish zooids, and transferred it to a glass bowl with sea water sterilized with UV-light and filtered through a 0.2 μm mesh (UVFSW). The bowl was placed under a stereomiscroscope with direct light and a digital thermometer to monitor the water temperature. Ripe colonies began to spawn in around 5 minutes or more, or usually when the temperature reached 15 °C. Once the temperature rose to 16 °C, the bowl was cooled down on ice with no direct light.

A spawning colony was sequentially transferred to new bowls with UVFSW at 10 °C to distribute the vast amounts of eggs. For each bowl with eggs, we added a solution of ethylenediaminetetraacetic acid (EDTA) to a final volume of 0.1 mM (usually ~20 μL of 0.5 M EDTA) to induce egg activation [52]. The bowl was then placed in a incubator at 15 °C for 30–60 min. Activated eggs were concentrated by swirling, distributed to smaller glass bowls with UVFSW, and washed twice to remove the EDTA. We adjusted the number of eggs per bowl so that eggs sitting on the bottom of the dish no longer touched each other. We kept the cultures at 15 °C. One colony could be re-used for spawning multiple times. *M. membranacea* colonies maintained in the flowing tanks remained viable to developmental studies for a week.

### 4D recordings and cell tracing

We pipetted embryos at the 2-cell stage to a glass slide coated with poly-L-lysine. We mounted the embryos under a cover slip with supporting clay feet, completed the volume with UVFSW, and sealed the cover slip with vaseline. The slide was put under an automated 4D microscopy system (Caenotec, r.schnabel@tu-bs.de) [167] with a cooling ring around the objective to keep the temperature at 15 °C. We recorded whole-embryo stacks (40–60 optical slices) every 40 s under differential interference contrast for four individuals (wt1–wt4). Development was recorded for approximately 24 h, until the embryos became ciliated and swam away from the field of view (Additional file 1: Video S1 and Additional file 2: Video S2).

We loaded the image sequence data into the tracking software Simi BioCell (Simi®) [168] and manually traced individual cells. We programmatically analyzed the cell lineage data using simi.py, a Python library we wrote for this purpose and available at https://github.com/nelas/simi.py. To conduct further analyses and visualization of the data, we used simi.py to convert the data from Simi BioCell into the format of MaMuT (Massive Multi-view Tracker) [169], a cell lineage tracker integrated with the BigDataViewer [170] for visualization of large image datasets on the Fiji open source platform [171].

The source cell lineage files, the processed and converted cell lineages, and the data files used for all analyses have been deposited in a data repository and are available for download [172].

### Cell lineage nomenclature

We annotated the individual cells of *M. membranacea* using the spiral cleavage nomenclature [6, 9, 11] with modifications to better describe the unique features of the bryozoan cell lineage. The blastomeres at the 4-cell stage were labeled as A, B, C, and D according to their fate, as identified by the video recordings. For example, the blastomere giving rise to the dorsal/posterior region of the cyphonautes larva was assigned to the D quadrant. The quartets derived from the four large blastomeres were labeled as in spiral cleavage (1q–4q). At the 8-cell stage, the animal blastomeres were named 1a–1d and the vegetal blastomeres 1A–1D. Where applicable, the progeny of a blastomere received the standard superscripts of spiral cleavage, with ^1^ for the apical daughter cell and ^2^ for the basal daughter cell. We have adapted the nomenclature to accommodate the first cleavage of the animal blastomeres, where cells occupy the same position in the animal–vegetal axis at the 16-cell stage. The four central cells received the subscript _i_ (for internal), and the four outer cells received the subscript _e_ (for external). As an example, the cell 1a_i_^1^ is the apical progeny of the internal A quadrant animal blastomere. If cells divided along the anteroposterior axis, they received the ^A^ or ^P^ superscript. In addition, we labeled the progeny of meridional divisions with the superscript ^R^, for the cell to the right, and ^L^, for the cell to the left when viewed from the animal pole.

### Fixation methods

We fixed representative developmental stages for antibody staining in 4% formaldehyde for 1 h at room temperature, washed the embryos in PTw (1x PBS + 0.1% Tween-20) and stored them in PTw at 4 °C. For in situ hybridization, we fixed the samples in a solution of 4% formaldehyde/0.2% glutaraldehyde solution to avoid tissue damage during the protocol. After 1 h fixation at room temperature, we washed the embryos in PTw, dehydrated them through a methanol series, and kept the samples in 100% methanol at –20 °C.

### Gene cloning and in situ hybridization

We assembled the Illumina RNA-seq reads from *M. membranacea* (NCBI SRA Project: SRX1121923) with Trinity [173] and used known genes to identify putative orthologs in the transcriptome. We performed PCR using gene-specific primer pairs on cDNA synthesized with the SMARTer RACE cDNA Amplification kit (Clontech). Primers were designed with Primer3 [174]. Gene sequences and the corresponding primer pairs were deposited in the GenBank (NCBI) with the accession numbers KY565381–KY565397, and are also available in the data repository [172]. We synthesized antisense DIG-labeled riboprobes with MEGAscript kit (Ambion) and performed colorimetric in situ hybridization according to an established protocol [70]. We observed no significant variability in the gene expression patterns between individual embryos.

### Gene orthology

Orthology was assigned by aligning amino acid sequences of *M. membranacea* against annotated genes from diverse metazoans using MAFFT 7.271 [175], retaining only informative portions of the alignment with GBlocks 0.91b with relaxed parameters [176] and running a Maximum Likelihood phylogenetic analysis with RAxML 8.2.4 [177] using automatic model recognition and rapid bootstrap. We manually verified the alignments using UGENE [178]. Resulting trees from the maximum likelihood analysis were rendered into cladograms using the ETE Toolkit [179] (Additional file 19: Figure S11). Gene orthology runs and source files are available in the data repository [172].

### Immunohistochemistry and MAPK antibody

We permeabilized the embryos with several washes in PTx (1x PBS + 0﻿.2% Triton X-100) for 2 h and blocked with two washes of 1 h in PTx + 0.1% bovine serum albumin (BSA), succeeded by 1 h incubation in PTx + 5% normal goat serum. Samples were incubated with the primary antibody for the MAPK diphosphorylated ERK-1&2 (Sigma M9692-200UL) diluted 1:200, and stored overnight at 4 °C on a nutator. We removed the MAPK antibody with three 5 min and four 30 min washes in PTx + 0.1% BSA, blocked in PTx + 5% normal goat serum for 1 h, and incubated nutating overnight at 4 °C with the secondary antibody Anti-Mouse-POD conjugate (Jackson) diluted 1:250. We removed the secondary antibody with three 5 min followed by several washes in PTx + 0.1% BSA for 2 h.

To amplify and develop the signal, we incubated the embryos for 3–5 min with the provided reagent solution and fluorochrome from TSA reagent kit Cy5 (Perkin Elmer). We stopped the reaction with two washes in a detergent solution (50% formamide, 2x SSC, 1% SDS) at 60 °C to reduce background, followed by PTw washes at room temperature. We stained nuclei by incubating permeabilized embryos in DAPI 1:500, Sytox Green 1:1000, or Propidium Iodide 1:500 for 2 h. Nuclei staining was combined with f-actin staining by the addition of BODIPY FL phallacidin 5 U/mL previously evaporated to remove methanol.

We repeated the immunostaining several times under different conditions to optimize the signal-to-noise ratio for different developmental stages to identify the developmental sequence of MAPK activation and using at least 100 embryos per well to account for individual variability.

To verify the identity of the single MAPK-activated blastomere – the embryo is still biradial at this stage – we carefully cross-checked cell lineage, in situ hybridization, and immunohistochemistry data. The relative timing of division between the 3Q blastomeres is highly consistent between the four *M. membranacea* embryos analyzed in this study. The blastomeres 3A and 3C divide first (in synchrony), the 3B divides next and, around 3.5 h later, the division of the 3D blastomere finally occurs. By analyzing MAPK immunostainings of closely timed developmental stages, we were able to stage the embryos and fully resolve identity of the 3Q blastomeres. In addition, we performed immunostaining after the in situ hybridization of *nk2.1* and verified that the MAPK-activated blastomere is opposite to the *nk2.1* territory (restricted to the B quadrant) (see [172]).

### MAPK inhibition

We blocked the MAPK pathway in *M. membranacea* by soaking the embryos with the MEK inhibitor U0126 (Promega) – a compound that inhibits the activation of the MAPK (ERK 1&2) by inhibiting the kinase activity of MAP Kinase Kinase (MAPKK or MEK 1/2) [98]. U0126 has been extensively used to study the MAPK role in spiralian development [54–60]. We resuspended U0126 in dimethyl sulfoxide (DMSO) to a stock concentration of 10 mM, aliquoted to smaller volumes and stored the samples at –20 °C for no more than a week. We diluted the stock solution to working concentrations using UVFSW and incubated the treated embryos. Controls were incubated in UVFSW and in DMSO diluted in UVFSW 1:400, i.e., the maximum amount of DMSO used in treated wells. The MAPK experiments were conducted at 10 °C usually in two setups, a fine-picked where only 2-cell embryos were collected to assure we were seeing the inhibitor effects in healthy embryos, and a course-picked sample where a larger amount of embryos was picked.

### Microscopy and image processing

We mounted in situ embryos in 70% glycerol in PTw. Embryos from antibody staining were mounted in 97% 2,2’-thiodiethanol [180, 181], 80% glycerol in PBS, or SlowFade® Gold Antifade (ThermoFisher). We imaged the samples with a Zeiss AxioCam HRc mounted on a Zeiss Axioscope A1, using differential interference contrast technique (Nomarski) for in situ hybridizations and a fluorescent lamp for the MAPK antibody staining. We used a Confocal Leica TCS SP5 to image fluorescent samples. Colorimetric in situ hybridizations were also scanned under the confocal using reflection microscopy [182]. We processed all resulting confocal stacks in Fiji [171]. When necessary, we adjusted the distribution of intensity levels to improve contrast with Fiji or GIMP. We created vector graphics and assembled the figure plates using Inkscape.

To convert the time-lapse image stacks from the 4D microscopy system to video, we blended the in-focus information from different focal planes (focus stacking) using the enfuse program of the Enblend/Enfuse software for each timepoint, and animated the resulting image sequence to 25 frames per second using FFmpeg. We exported the cell models generated by Simi BioCell and MaMuT and overlaid in the videos to show the position of the tracked cells in the embryo. The code for the image/video processing steps are available in our previous study [172].

## Additional files

Additional file 3: Figure S1. Overview of the cell tracking data of four wild type embryos of *M. membranacea*. (A) Raw cell lineages tracked in Simi BioCell [168]. wt1 is a recording of the animal pole providing most of the data for the aboral epithelium, wt2 is a vegetal pole view providing detailed information for the vegetal ectoderm, and wt3 and wt4 are additional recordings of the animal pole. Development time measured in hours post activation (hpa) is shown in the Y axis. (B) Number of tracked cells per embryo showing the proportion of cells by quadrant. wt1 is the most-complete cell lineage and the embryo with best coverage of the B quadrant. (C) Relative density of tracked cells per time for each embryo. The plot is complementary to the raw lineages and show that embryos wt1 and wt2 were tracked for a longer period than wt3 and wt4. The peaks indicate the moment that the maximum number of tracked cells was reached and when cell births begin to decrease, which is an indicator for the increase in the number of untracked cells. The data and code for generating the plots (B) and (C) are available at [172]. (PNG 2314 kb)

Additional file 4: Figure S2. Cell lineage variability in *M. membranacea*. (A) Overlap between the cell lineages of embryos wt1 to wt4 (*left*) and the birth time of individual cells up to 11 hpa (*right*). Only cells tracked in the four embryos are shown. The black horizontal lines indicate the mean birth time of a cell between the embryos, and vertical black lines the standard error. The embryo wt2 lags behind the other three embryos, but the variability in the timing of cell divisions is low. (B) Standard deviation for the birth time of a cell between different embryos by time of development. The timing of cell divisions between homologous cells does not surpass 20 min until 9 hpa. After that, the variability increases. (C) Same as (B) but only for embryos tracked from the animal pole (wt1, wt3, and wt4). Timing variability also increases over time, but the range of variation is contained within 20 min even after 9 hpa, values that are in the same order of magnitude of the variability observed in *C. elegans* [168]. The data and code for generating the plots are available at [172]. (PNG 420 kb)

Additional file 6: Figure S3. Quartet synchrony in animal pole embryos of *M. membranacea*. Color gradient represents the time a cell took to divide after the first cell of its quartet had divided. Each column represents one embryo that has been tracked from the animal pole (wt1, wt3, and wt4). Quartets 1q_i_^12^ and 1q_e_^11^ show a consistent asynchronous event between the three embryos. Raw data, code to generate the plot, and additional comparative plots including all embryos are available at [172]. (PNG 52 kb)

Additional file 8: Figure S4. Detailed fate map of *M. membranacea*. The data reflect the consensus between embryos wt1, wt2, wt3, and wt4. Quadrant A, B, C, and D give rise to the left, anterior, right, and posterior regions of the embryo. Question marks indicate cells whose fate could not be determined. (PNG 972 kb)

Additional file 10: Figure S5. Sequence of cell divisions in the vegetal ectoderm of wt2. Images from Additional file 9: Video S5, but oriented with the D quadrant to the bottom and mirrored due to the reverse chirality of this embryo (see [53]). (A) 28-cell stage (6.7 hpa) showing the quartets 2q (*purple*), 3q (*blue*), and 3Q (*black*). (B) The second quartet divides forming the founders of the vegetal 12-tet (*blue*). (C) The 3Q quartet begins to be internalized. White dashed line demarcates the blastoporal lip. (D) Second generation of outer (*blue*) and central (*yellow*) 12-tets. (E) Eleven of the 12 cells have divided (*yellow*) and surround the 4Q cells (*black*). The cell 3c divides later than the others. This delay was only observed in embryo wt2 and might indicate a developmental variability between embryos (see synchrony plots in [172]). (F) After 3c divides, the blastopore (*white dashed line*) is demarcated by 12 cells from the same generation. (G) The cells lining the blastopore begin to divide forming central (*red*) cells. Some of the outer (*blue*) cells also begin to divide (*green*). (H) The blastopore is now demarcated by seven cells third generation (*red*) and 3c^2^ from the second generation (*yellow*). The blastopore is now narrower. (I) The cells at the vertices of the second generation (*yellow*) divide forming cells at the vertices of the embryo that are not part of the blastoporal lip. (PNG 2522 kb)

Additional file 12: Figure S6. Development of *M. membranacea* under different concentrations of the MEK inhibitor U0126. (A) Maximum intensity projection of a confocal stack for the most representative phenotype of each U0126 treatment. Ratio in the lower left corner shows the number of embryos scored for the shown phenotype versus the total number of embryos in the treatment. Phenotypes in an additional seawater-only treatment (no DMSO) were indistinguishable from DMSO control and had a ratio of 31/37 (not shown). (B) Measurements for the number of nuclei (y axis), embryo width (point size), and embryo height (color scale) for the confocal scans of (A). Each colored point represents one embryo, the black rhombus and error bars shows the mean number of nuclei with standard error. Number of embryos scanned and measured per treatment: DMSO = 3, 1 μM = 3, 10 μM = 5, 25 μM = 4. In all treatments, U0126 was added at 3 hpa (2-cell stage), the embryos developed at 10 °C and were fixed at 44 hpa. Scale bars = 20 μm. Raw data and code to generate the plot are available at [172]. (PNG 829 kb)

Additional file 13: Figure S7. *M. membranacea* embryos treated with the MEK inhibitor U0126 (10 μM) from different developmental stages. All treatments developed at 10 °C and were fixed at 72 hpa. *Arrowhead* indicates when the U0126 treatments began for each experimental condition, represented by the horizontal colored lines. Representative phenotypes are shown for the 4, 8, and 18 hpa treatments. We scored 100 embryos under light microscopy for each treatment to obtain the ratio of severe/mild phenotypes. Treatments showing the severe phenotype are shown in *orange*, with the percentage of severe phenotypes indicated at the right end. Treatments without severe phenotypes were colored in *yellow*. (PNG 865 kb)

Additional file 14: Figure S8. 4D recordings of *M. membranacea* embryos treated with the MEK inhibitor U0126. Each row corresponds to the timeline of an individual embryo. All recordings were synchronized by the timing of the second cleavage (4-cell stage = 0 h). Time scale shows the number of hours after 4-cell stage. The exact developmental time is shown on panels that do not correspond to the time shown in the main scale (*top right corner*). Frame number of each panel is shown on the *bottom left* corner (a frame was captured every 40 s). The orange rectangle indicates the cleavage abnormality observed in embryos that exhibit the severe phenotype. (PNG 6748 kb)

Additional file 15: Figure S9. Gene expression throughout *M. membranacea* cell lineage. (A) Various developmental stages illustrating the gene expression patterns in the animal ectoderm, vegetal ectoderm, and endomesoderm with cellular resolution. (B) Cell lineage diagrams indicating the lineages where the above genes are expressed. Vivid colors indicate gene expression while more transparent branches indicate absence of expression for each particular gene analyzed. (PNG 1047 kb)

Additional file 16: Table S1. MAPK activity in spiralians. Based on [54–60, 99]. (PDF 77 kb)

Additional file 17: Figure S10. Cell lineage comparison between the larval ciliated bands of *M. membranacea* and *Patella vulgata* [106]. (PNG 139 kb)

Additional file 18: Table S2. Gene expression patterns in spiralians. Based on [57, 63, 67, 68, 71, 79, 80, 86, 87, 99, 117–123, 129, 132, 137, 142–150, 154–156, 187–216]. (PDF 72 kb)

Additional file 19: Figure S11. Orthology assignment for the bryozoan genes used in this study. (A) *six3/6*, (B) *dlx* and *evx*, (C) *otx* and *gsc*, (D) *pax6*, (E) *nk2.1*, (F) *foxa*, *foxc*, and *foxf*, (G) *nanos*, (H) *bra*, (I) *cdx*, (J) *twist*, (K) *gata456*, and (L) *wnt1*. Cladograms show branch support values and bryozoan orthologs in *red*. (PDF 3134 kb)

## Abbreviations

DMSO: dimethyl sulfoxide
dpa: days post activation
EDTA: ethylened iami netetraacetic acid
hpa: hours post activation
PBS: phosphate-bu ffered saline
UVFSW: filtered sea water sterilized with UV light
