# Supplementary figures and images for: Cleavage modification did not alter blastomere fates during bryozoan evolution
Source: BMC Biol. 2017 Apr 28;15:33. doi: 10.1186/s12915-017-0371-9 (PMC5408385; doi:10.1186/s12915-017-0371-9)

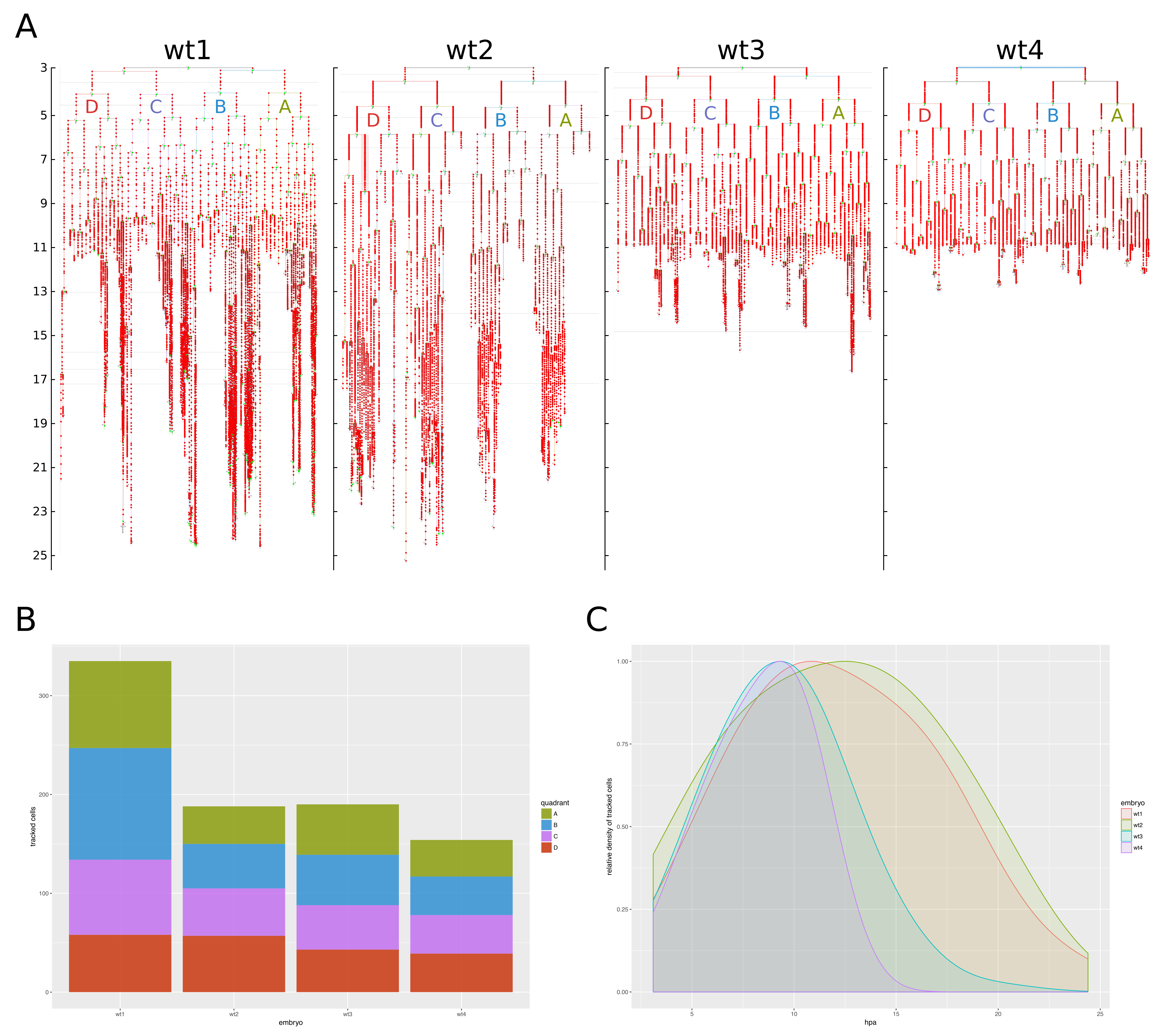

Supplement: Supplementary file 7 — Overview of the cell tracking data of four wild type embryos of M. membranacea. (A) Raw cell lineages tracked in Simi BioCell [168]. wt1 is a recording of the animal pole providing most of the data for the aboral epithelium, wt2 is a vegetal pole view providing detailed information for the vegetal ectoderm, and wt3 and wt4 are additional recordings of the animal pole. Development time measured in hours post activation (hpa) is shown in the Y axis. (B) Number of tracked cells per embryo showing the proportion of cells by quadrant. wt1 is the most-complete cell lineage and the embryo with best coverage of the B quadrant. (C) Relative density of tracked cells per time for each embryo. The plot is complementary to the raw lineages and show that embryos wt1 and wt2 were tracked for a longer period than wt3 and wt4. The peaks indicate the moment that the maximum number of tracked cells was reached and when cell births begin to decrease, which is an indicator for the increase in the number of untracked cells. The data and code for generating the plots (B) and (C) are available at [172]. (PNG 2314 kb) [file 12915_2017_371_MOESM3_ESM.png]

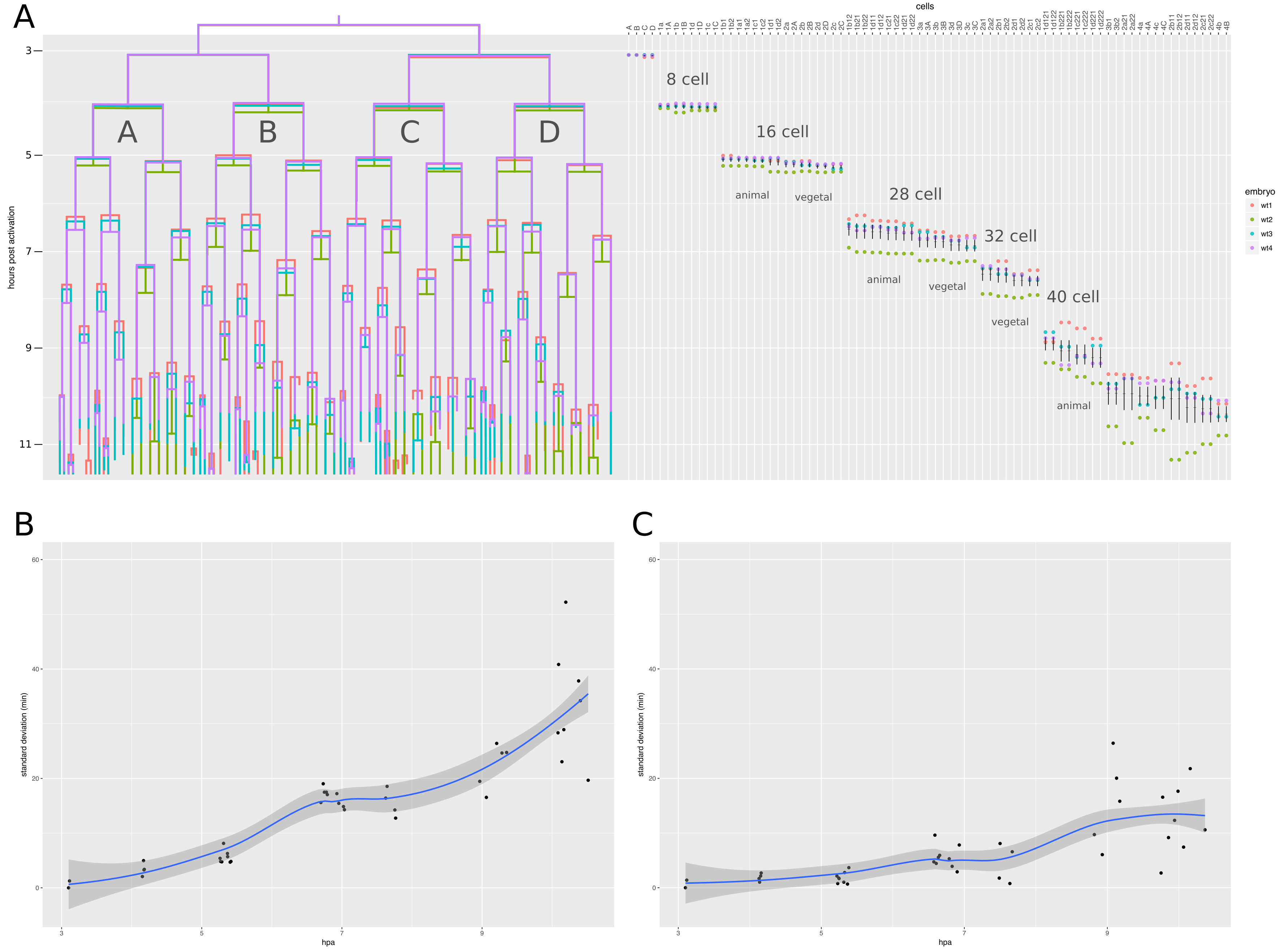

Supplement: Supplementary file 8 — Cell lineage variability in M. membranacea. (A) Overlap between the cell lineages of embryos wt1 to wt4 (left) and the birth time of individual cells up to 11 hpa (right). Only cells tracked in the four embryos are shown. The black horizontal lines indicate the mean birth time of a cell between the embryos, and vertical black lines the standard error. The embryo wt2 lags behind the other three embryos, but the variability in the timing of cell divisions is low. (B) Standard deviation for the birth time of a cell between different embryos by time of development. The timing of cell divisions between homologous cells does not surpass 20 min until 9 hpa. After that, the variability increases. (C) Same as (B) but only for embryos tracked from the animal pole (wt1, wt3, and wt4). Timing variability also increases over time, but the range of variation is contained within 20 min even after 9 hpa, values that are in the same order of magnitude of the variability observed in C. elegans [168]. The data and code for generating the plots are available at [172]. (PNG 420 kb) [file 12915_2017_371_MOESM4_ESM.png]

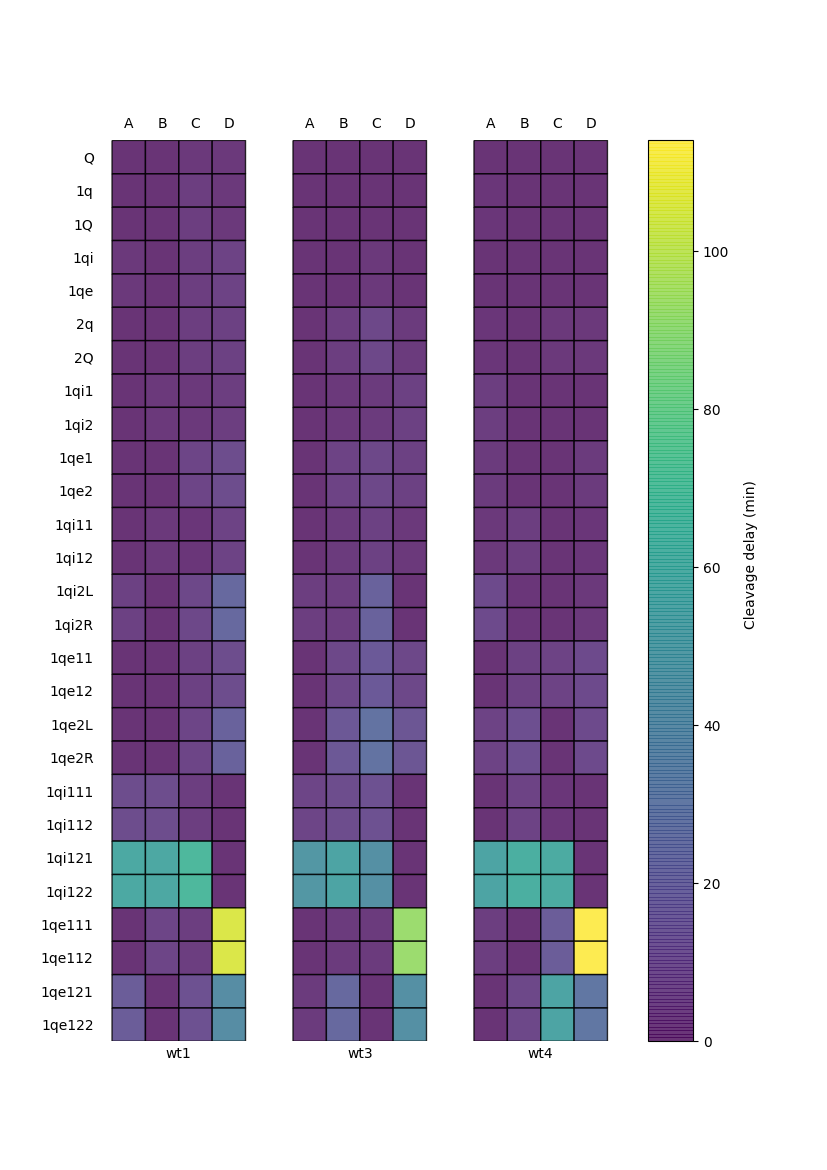

Supplement: Supplementary file 9 — Quartet synchrony in animal pole embryos of M. membranacea. Color gradient represents the time a cell took to divide after the first cell of its quartet had divided. Each column represents one embryo that has been tracked from the animal pole (wt1, wt3, and wt4). Quartets 1qi 12 and 1qe 11 show a consistent asynchronous event between the three embryos. Raw data, code to generate the plot, and additional comparative plots including all embryos are available at [172]. (PNG 52 kb) [file 12915_2017_371_MOESM6_ESM.png]

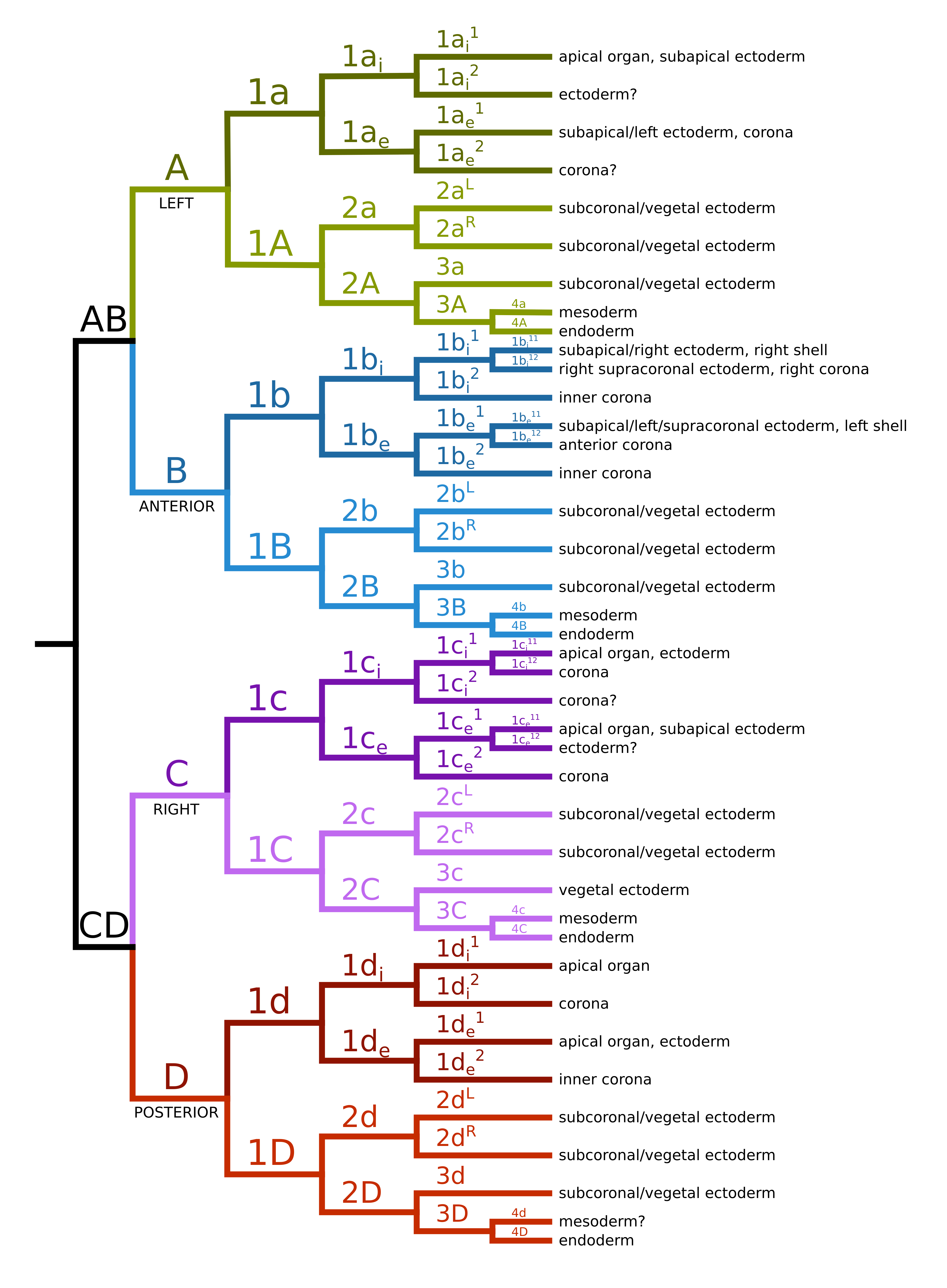

Supplement: Supplementary file 10 — Detailed fate map of M. membranacea. The data reflect the consensus between embryos wt1, wt2, wt3, and wt4. Quadrant A, B, C, and D give rise to the left, anterior, right, and posterior regions of the embryo. Question marks indicate cells whose fate could not be determined. (PNG 972 kb) [file 12915_2017_371_MOESM8_ESM.png]

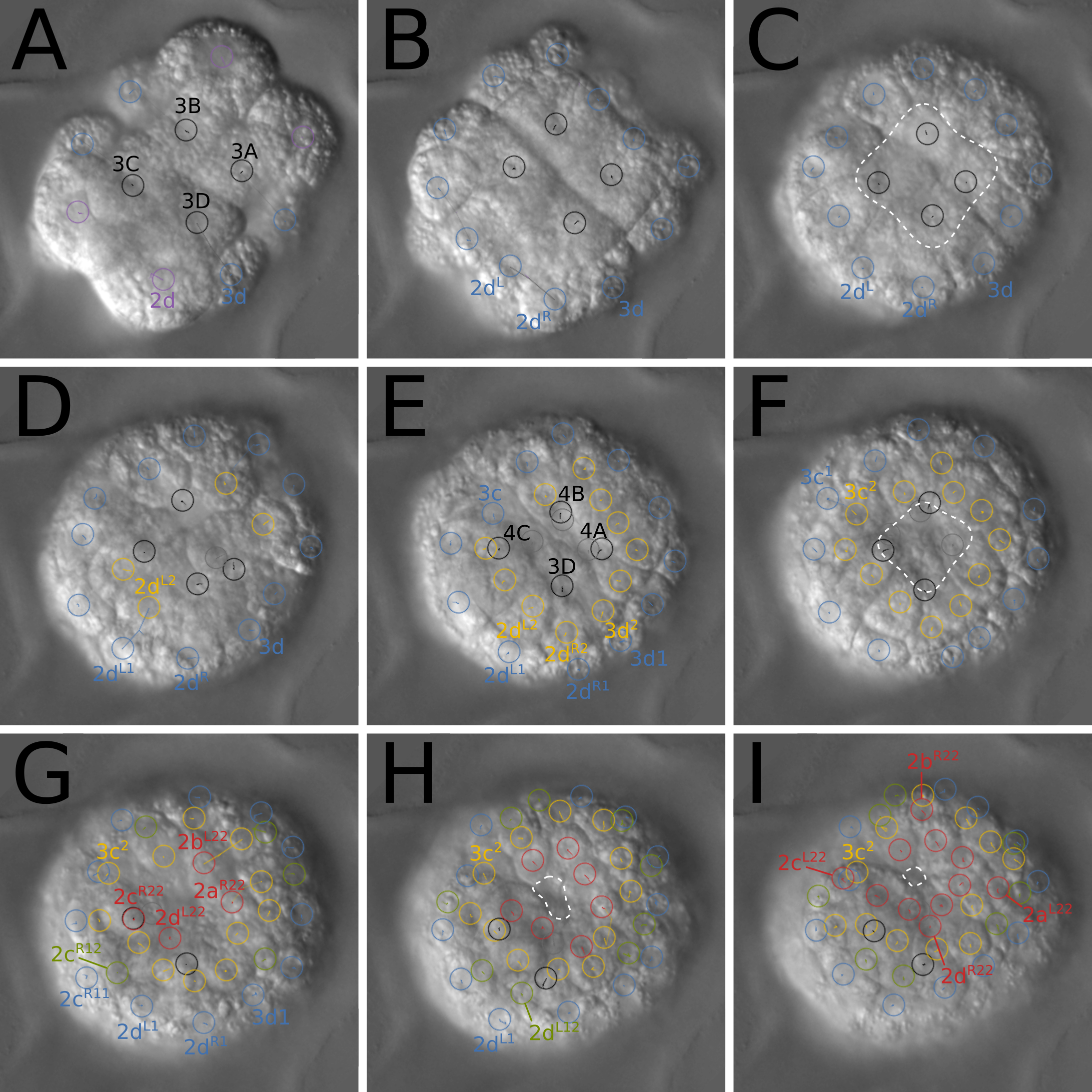

Supplement: Supplementary file 11 — Sequence of cell divisions in the vegetal ectoderm of wt2. Images from Additional file 9: Video S5, but oriented with the D quadrant to the bottom and mirrored due to the reverse chirality of this embryo (see [53]). (A) 28-cell stage (6.7 hpa) showing the quartets 2q (purple), 3q (blue), and 3Q (black). (B) The second quartet divides forming the founders of the vegetal 12-tet (blue). (C) The 3Q quartet begins to be internalized. White dashed line demarcates the blastoporal lip. (D) Second generation of outer (blue) and central (yellow) 12-tets. (E) Eleven of the 12 cells have divided (yellow) and surround the 4Q cells (black). The cell 3c divides later than the others. This delay was only observed in embryo wt2 and might indicate a developmental variability between embryos (see synchrony plots in [172]). (F) After 3c divides, the blastopore (white dashed line) is demarcated by 12 cells from the same generation. (G) The cells lining the blastopore begin to divide forming central (red) cells. Some of the outer (blue) cells also begin to divide (green). (H) The blastopore is now demarcated by seven cells third generation (red) and 3c2 from the second generation (yellow). The blastopore is now narrower. (I) The cells at the vertices of the second generation (yellow) divide forming cells at the vertices of the embryo that are not part of the blastoporal lip. (PNG 2522 kb) [file 12915_2017_371_MOESM10_ESM.png]

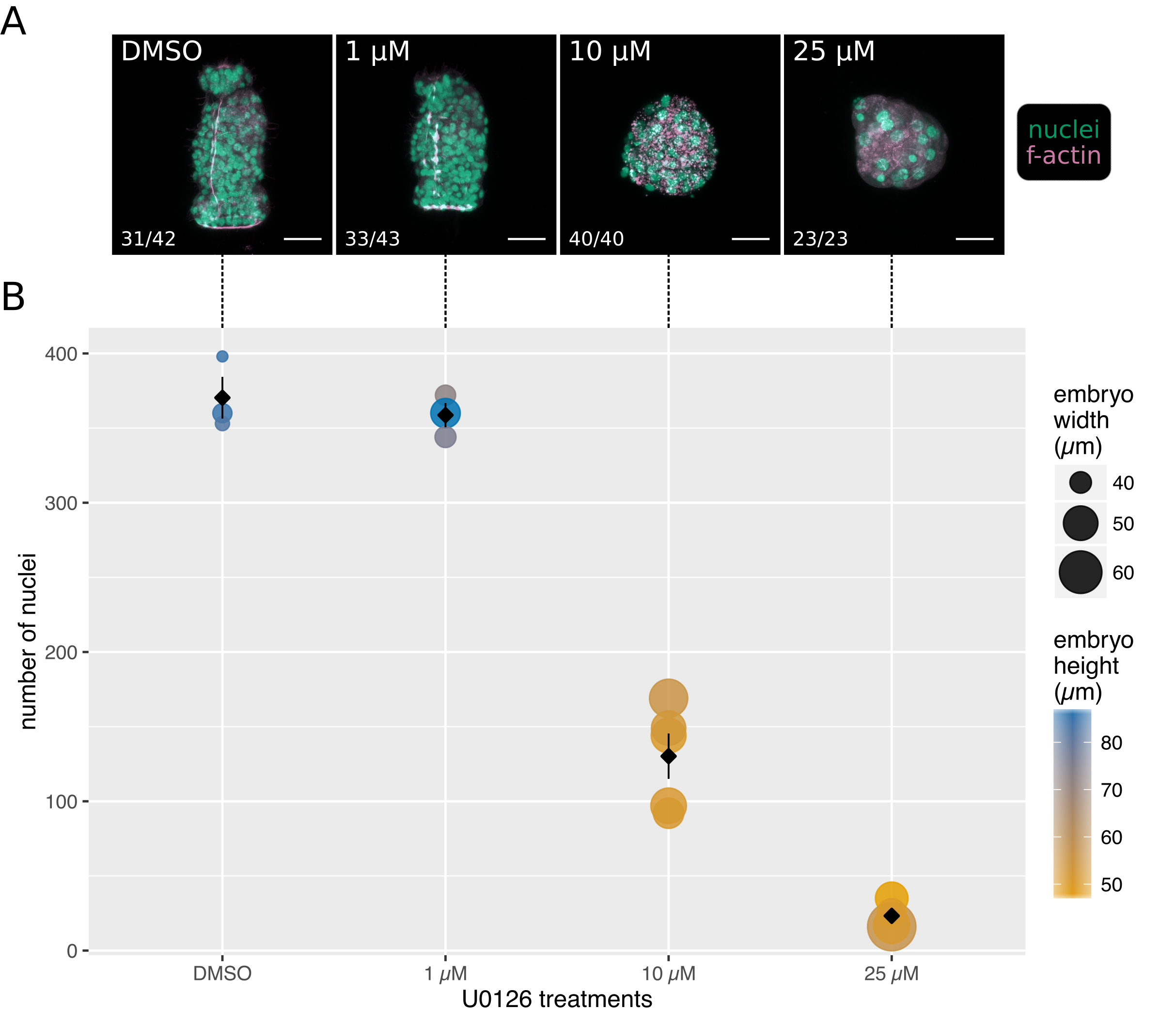

Supplement: Supplementary file 12 — Development of M. membranacea under different concentrations of the MEK inhibitor U0126. (A) Maximum intensity projection of a confocal stack for the most representative phenotype of each U0126 treatment. Ratio in the lower left corner shows the number of embryos scored for the shown phenotype versus the total number of embryos in the treatment. Phenotypes in an additional seawater-only treatment (no DMSO) were indistinguishable from DMSO control and had a ratio of 31/37 (not shown). (B) Measurements for the number of nuclei (y axis), embryo width (point size), and embryo height (color scale) for the confocal scans of (A). Each colored point represents one embryo, the black rhombus and error bars shows the mean number of nuclei with standard error. Number of embryos scanned and measured per treatment: DMSO = 3, 1 μM = 3, 10 μM = 5, 25 μM = 4. In all treatments, U0126 was added at 3 hpa (2-cell stage), the embryos developed at 10 °C and were fixed at 44 hpa. Scale bars = 20 μm. Raw data and code to generate the plot are available at [172]. (PNG 829 kb) [file 12915_2017_371_MOESM12_ESM.png]

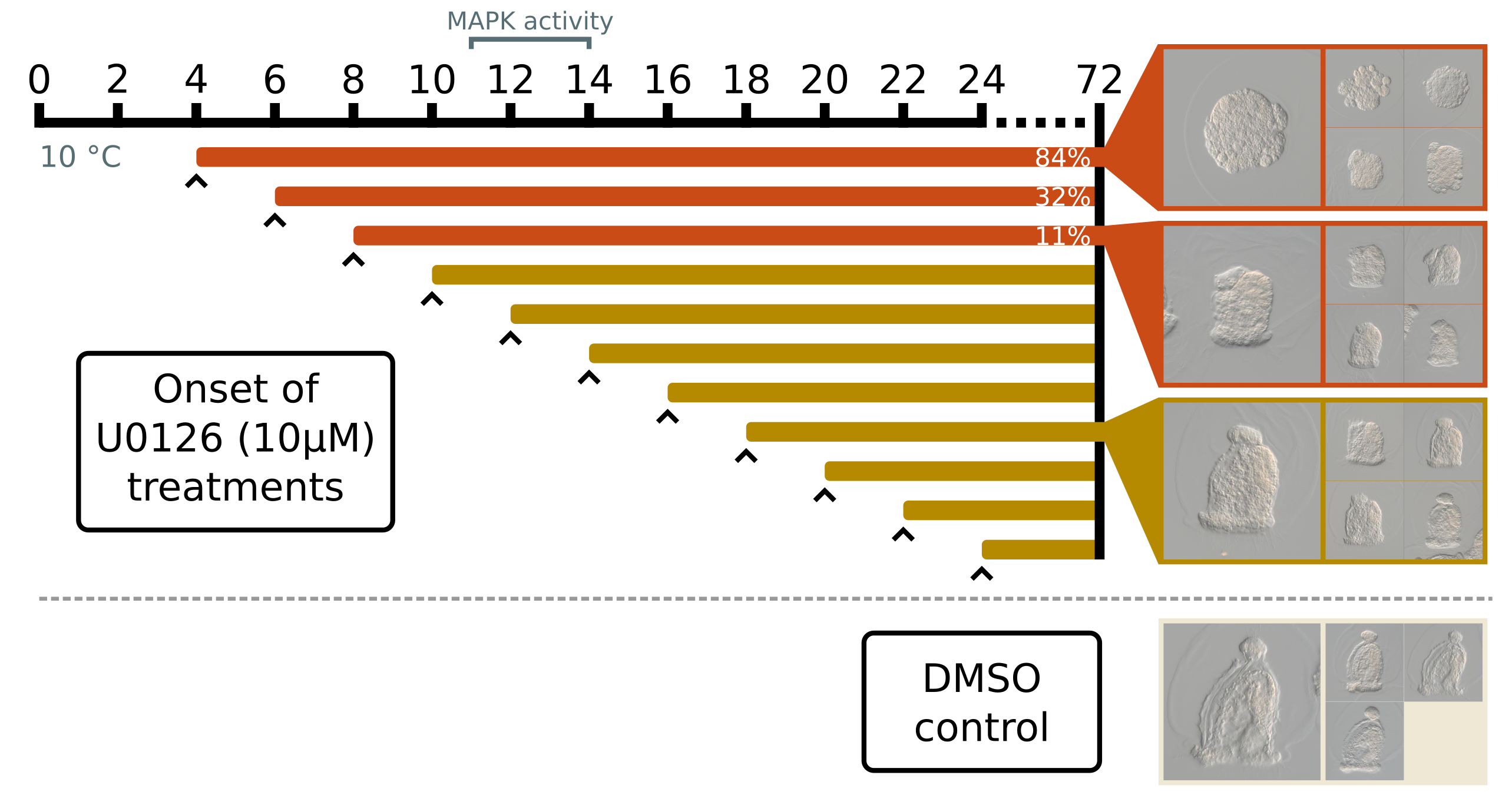

Supplement: Supplementary file 13 — M. membranacea embryos treated with the MEK inhibitor U0126 (10 μM) from different developmental stages. All treatments developed at 10 °C and were fixed at 72 hpa. Arrowhead indicates when the U0126 treatments began for each experimental condition, represented by the horizontal colored lines. Representative phenotypes are shown for the 4, 8, and 18 hpa treatments. We scored 100 embryos under light microscopy for each treatment to obtain the ratio of severe/mild phenotypes. Treatments showing the severe phenotype are shown in orange, with the percentage of severe phenotypes indicated at the right end. Treatments without severe phenotypes were colored in yellow. (PNG 865 kb) [file 12915_2017_371_MOESM13_ESM.png]

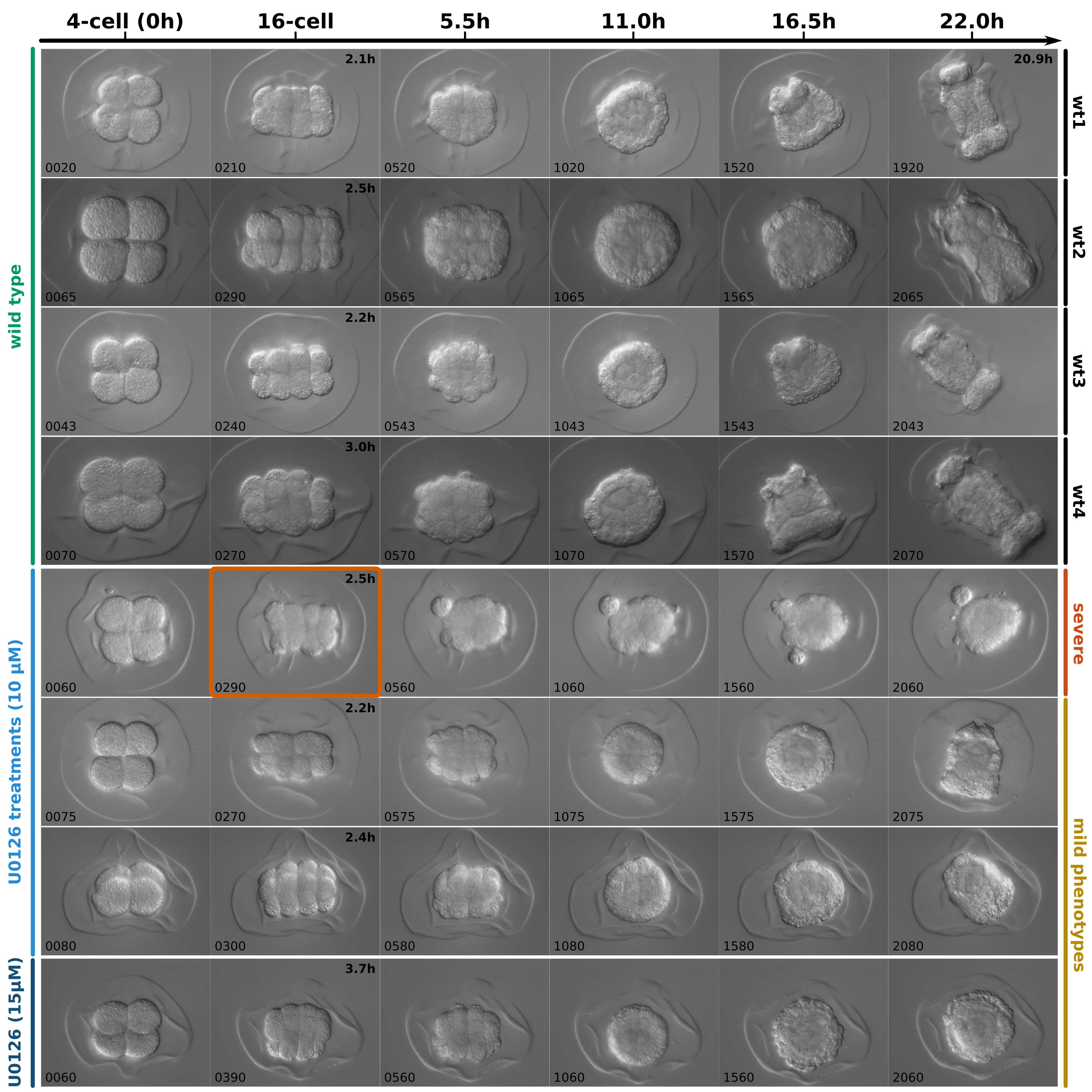

Supplement: Supplementary file 14 — 4D recordings of M. membranacea embryos treated with the MEK inhibitor U0126. Each row corresponds to the timeline of an individual embryo. All recordings were synchronized by the timing of the second cleavage (4-cell stage = 0 h). Time scale shows the number of hours after 4-cell stage. The exact developmental time is shown on panels that do not correspond to the time shown in the main scale (top right corner). Frame number of each panel is shown on the bottom left corner (a frame was captured every 40 s). The orange rectangle indicates the cleavage abnormality observed in embryos that exhibit the severe phenotype. (PNG 6748 kb) [file 12915_2017_371_MOESM14_ESM.png]

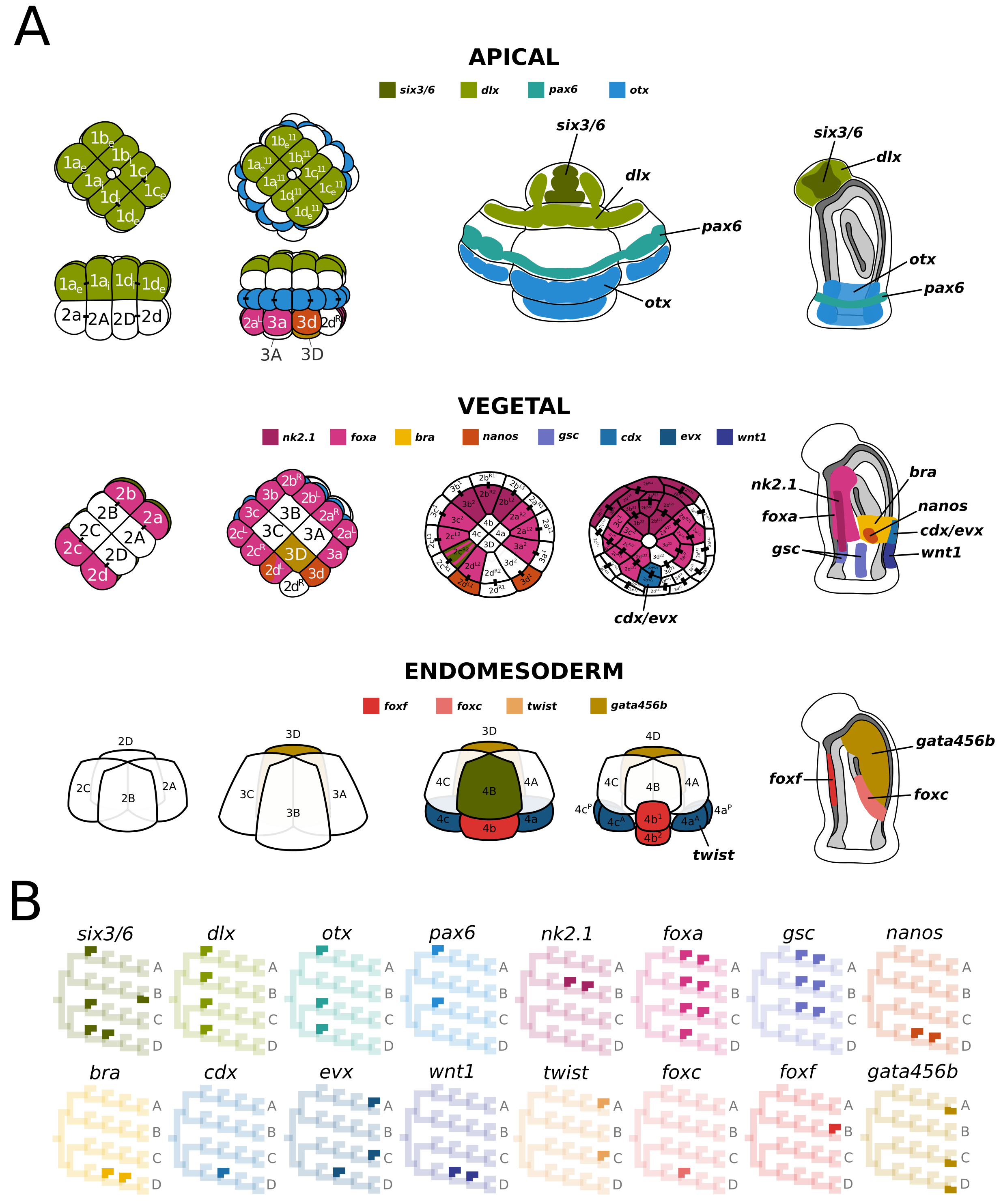

Supplement: Supplementary file 15 — Gene expression throughout M. membranacea cell lineage. (A) Various developmental stages illustrating the gene expression patterns in the animal ectoderm, vegetal ectoderm, and endomesoderm with cellular resolution. (B) Cell lineage diagrams indicating the lineages where the above genes are expressed. Vivid colors indicate gene expression while more transparent branches indicate absence of expression for each particular gene analyzed. (PNG 1047 kb) [file 12915_2017_371_MOESM15_ESM.png]

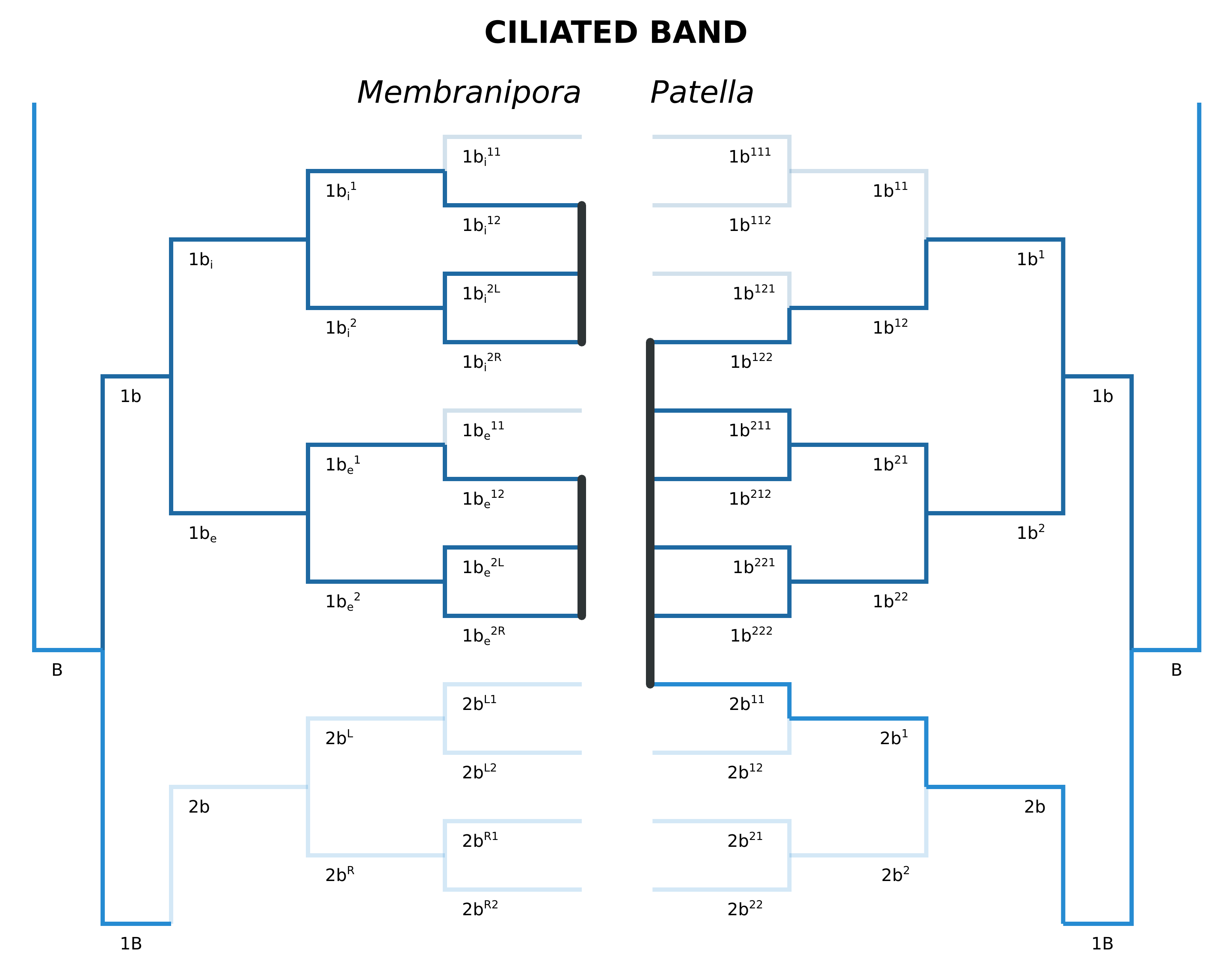

Supplement: Supplementary file 17 — Cell lineage comparison between the larval ciliated bands of M. membranacea and Patella vulgata [106]. (PNG 139 kb) [file 12915_2017_371_MOESM17_ESM.png]

A

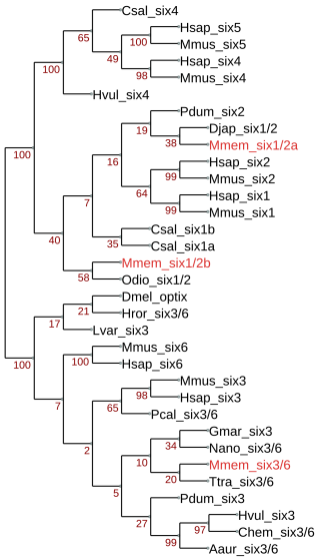

**B**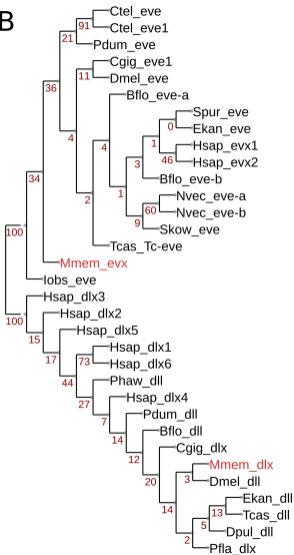

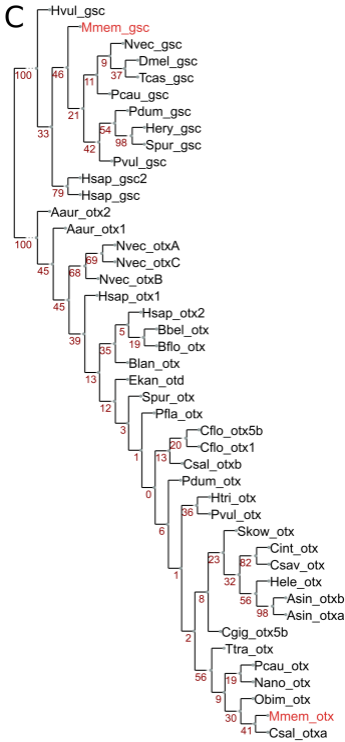

D

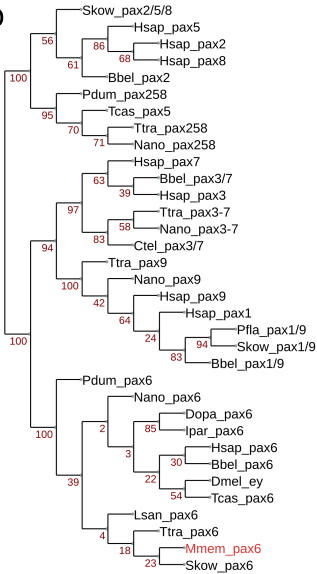

**E**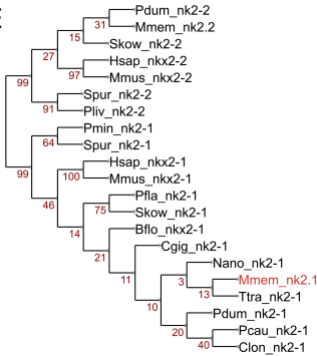

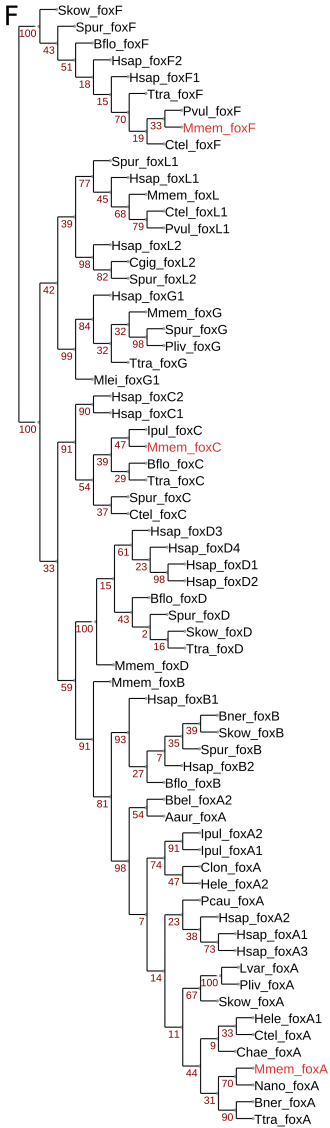

G

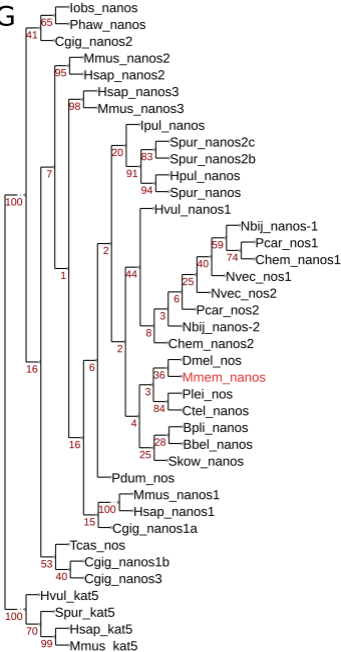

H

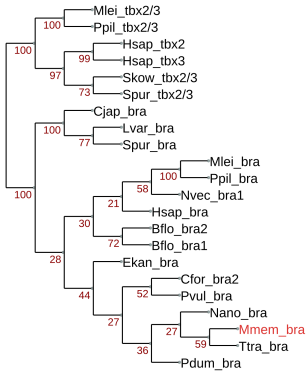

I

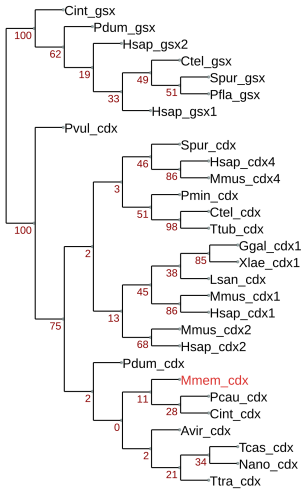

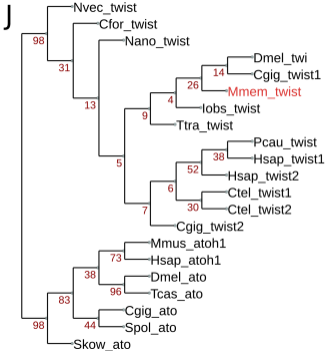

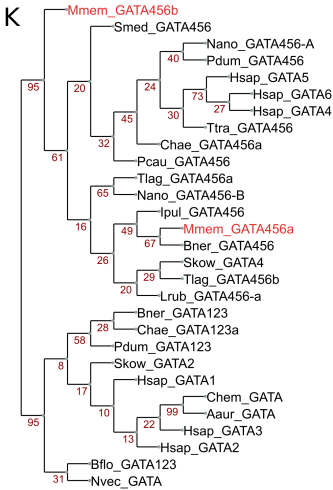

L

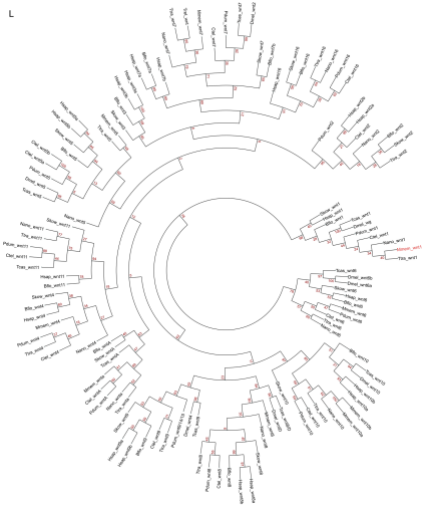

Supplement: Supplementary file 19 — Orthology assignment for the bryozoan genes used in this study. (A) six3/6, (B) dlx and evx, (C) otx and gsc, (D) pax6, (E) nk2.1, (F) foxa, foxc, and foxf, (G) nanos, (H) bra, (I) cdx, (J) twist, (K) gata456, and (L) wnt1. Cladograms show branch support values and bryozoan orthologs in red. (PDF 3134 kb) [file 12915_2017_371_MOESM19_ESM.pdf]
