# Supplementary material for: Cleavage modification did not alter blastomere fates during bryozoan evolution
Source: BMC Biol. 2017 Apr 28;15:33. doi: 10.1186/s12915-017-0371-9 (PMC5408385; doi:10.1186/s12915-017-0371-9)
Supplement: Supplementary file 16 — MAPK activity in spiralians. Based on [54–60, 99]. (PDF 77 kb) [file 12915_2017_371_MOESM16_ESM.pdf]

Table 1. MAPK activity in spiralians.

taxon	species	ABCD sizes	MAPK activity	MAPK inhibition (U0126)
Mollusca (gastropod)	*Ilyanassa obsoleta*	Unequal	Strong signal in the cytoplasm around nucleus of the 3D cell. Activity spread to the overlying 2d1 and $2d^2$; then $1c^{12}$, $1a^{12}$, $1b^{12}$; 4d cell, but not 4D; derivatives of the second and third quartet forming an arc centered in the dorsal midline; micromeres $2b^{12}$ and $3b^1$ transiently; undetectable 6h after 4d formation; never detected in 3A, 3B, 3C, $2b^2$ or 3a [54].	10µM at 23°C – Disorganized and incomplete larvae. MAPK activation is required in the micromeres themselves, not only in 3D. Alters the cleavage pattern in the third quartet of micromeres and timing of division of the 4d [54].
Mollusca (gastropod)	*Crepidula fornicata*	Equal-like	$1a^1$-$1d^1$ micromeres, fainter in $1a^2$-$1d^2$; 3D cell, persisting in 4d; also $3d^1$-$3d^2$ and $1a^{12}$-$1d^{12}$ [56].	10–25µM at 20–24°C – Radialized embryos with no differentiated axial properties or cell fates [56].
Mollusca (gastropod)	*Stagnicola palustris* (=*Lymnaea palustris*)*	Equal	3D only [55].	-
Mollusca (gastropod)	*Lottia scutum* (=*Tectura scutum*)*	Equal	3D only [55].	25–50µM at 14°C – Prevented normal development of micromeres producing the foot, the shell and affecting the differentiation of eyes [55].
Mollusca (gastropod)	*Haliotis asinina*	Equal	3D only [57].	10–50µM at 25°C – Retarded development and failure to develop larval musculature, shell and foot. But gene expression not completely radialized and treated embryos at lower concentrations were only mildly abnormal [57].
Mollusca (gastropod)	*Patella vulgata*	Equal	3D only [99].	10–50µM at ~14°C – Overall morphology somewhat disturbed, but without gross axial or gastrulation defects [99].
Mollusca (polyplacophoran)	*Chaetopleura apiculata*	Equal	3D only [55].	-
Annelida (sedentary polychaete)	*Hydroides dianthus* (=*Hydroides hexagonus*)*	Equal	4d only [55].	-

taxon	species	ABCD sizes	MAPK activity	MAPK inhibition (U0126)
Annelida (sedentary polychaete)	*Capitella teleta*	Unequal	Cells around the blastopore during gastrulation [58].	5–50µM at 14°C – Compact shaped larva with less muscles and disorganized axon tracts, but without affecting the D quadrant specification, organizing activity or establishment of larval body axes [58].
Annelida (errant polychaete)	*Platynereis dumerilii*	Unequal	Two dorsal cells (nephroblasts) and micromeres around the blastopore [59].	10–50µM at 18°C – Shortened overall morphology with disorganized muscle and neural tracts [59].
Annelida (errant polychaete)	*Alitta virens*	Unequal	8-cell stage at 1c, 1d and 1D; all micromeres at 16-cell (no vegetal activity); 3d and 3D activity, off in micromeres; then 1q1 and 2d descendant, 3c, 3d, 3D. Fades after that [60].	40µM at 14°C – Motile larvae with normal bilateral symmetry, mesoderm present but different phenotypes [60].
Bryozoa (gymnolaemate)	*Membranipora membranacea*	Equal	3D only.	1–25µM at 10–15°C – Delayed shortened larvae.

*Synonyms taken from WoRMS database.
