# Supplementary material for: Cleavage modification did not alter blastomere fates during bryozoan evolution
Source: BMC Biol. 2017 Apr 28;15:33. doi: 10.1186/s12915-017-0371-9 (PMC5408385; doi:10.1186/s12915-017-0371-9)
Supplement: Supplementary file 18 — Gene expression patterns in spiralians. Based on [57, 63, 67, 68, 71, 79, 80, 86, 87, 99, 117–123, 129, 132, 137, 142–150, 154–156, 187–216]. (PDF 72 kb) [file 12915_2017_371_MOESM18_ESM.pdf]

Additional file 18: Table S2. Gene expression patterns in spiralians.

taxon	*six3/6*	*dlx*	*otx*	*pax6*	*nk2.1*	*foxa*	*gsc*	*nanos*
Rotifera	-	-	-	anterior bilateral patches [187]	-	-	-	oocyte and posterior patch of ovary cells [188]
Micrognathozoa	-	-	-	-	-	-	-	-
Gnathostomulida	-	-	-	-	-	-	-	-
Platyhelminthes	brain branches [117]	brain, eyes, head and pharynx [189]	brain [67]	eyes [190]	parenchyma [191]	pharynx duct [192]	central nervous system [193]	germline and eye precursors [194, 195]
Gastrotricha	-	-	-	-	-	-	-	-
Mollusca	anterior blastopore lip, apical ganglia and ocelli [122]	all animal pole cells and general ectoderm [196]	prototroch [123]; blastopore lip, ventral midline, mouth, mesoderm and velum [122]	optic region, brain and arms [197]	anterior most region of the stomodeum, crescent above the mouth [122]	blastopore lips, endoderm and mesoderm [122, 137]	anterior mesoderm during gastrulation, foregut and mantle edge in larval stages [137]; ecto and endomesoderm and anterior of blastopore lip [122]	animal pole cells then two posterior bilaterally symmetrical cells [198]; or 4d [199]

**taxon**	***six3/6***	***dlx***	***otx***	***pax6***	***nk2.1***	***foxa***	***gsc***	***nanos***
Annelida	apical domain encircling apical organ [63, 71]	apical ectoderm, apical organ, prototroch and ganglia [200]	early apical organ, foregut and prototroch [63, 68, 71], also brain and posterior growth zone [201]	anterior bilateral patches [202–204]	apical ventral domain above prototroch [71] and bilateral domains flanking blastopore [201]	vegetal plate ring with cleared posterior expression, foregut and hindgut [79, 80, 154, 201]	foregut and bilateral neural domains [68, 201]	mesodermal posterior growth zone [205]; anterior ectodermal clusters, foregut, posterior segmental zone [142]; ectodermal precursor [206]
Nemertea	vicinity of apical organ and ciliated band, cephalic imaginal discs, anterior invagination and apical disc [119, 120]; anterior cephalic imaginal discs, cephalic lobes and mouth [121]	-	around blastopore, anterior cephalic imaginal discs, anterior region and gut [121]	-	-	mouth and pharynx, also head and posterior [121]	pair of bilateral clusters near the blastopore and anterolateral in juvenile [121]	-
Brachiopoda	apical domain, endoderm [118, 129]	-	apical domain, foregut, endoderm [129, 207]	apical lobe [207, 208]	apical domain, foregut [118, 129]	foregut, endoderm [129]	apical domain, foregut [129]	-
Phoronida	-	-	-	-	-	-	-	-
Entoprocta	-	-	-	-	-	-	-	-

taxon	*six3/6*	*dlx*	*otx*	*pax6*	*nk2.1*	*foxa*	*gsc*	*nanos*
Bryozoa	apical organ, anterior endoderm and lateral vegetal plate cell	apical organ	corona, apical disc during gastrulation	bilateral domains above the corona	vegetal anterior domain, foregut	vegetal ectoderm around blastopore, foregut	two anterior vegetal cells and pair of bilateral domains	two posterior cells on the vegetal plate

Additional file 18: Table S2 (continued). Gene expression patterns in spiralians.

taxon	*bra*	*cdx*	*evx*	*wnt1*	*twist*	*foxc*	*foxf*	*gata456*
Rotifera	-	-	-	-	-	-	-	-
Micrognathozoa	-	-	-	-	-	-	-	-
Gnathostomulida	-	-	-	-	-	-	-	-
Platyhelminthes	-	-	-	posterior end [209, 210]	pharynx muscle [192]	-	-	blind gut [193]
Gastrotricha	-	-	-	-	-	-	-	-
Mollusca	3D and mostly posterior edge of the blastopore [99, 122]; posterior of blastopore [57]	posterior ectoderm [122, 143, 149], mesoderm close to the blastopore [122, 143] and mouth/esophagus [122]	-	-	anterior mesoderm but not in its mesenchymal progeny [122, 211]	mantle, foregut and trunk mesoderm [86]	bilateral mesodermal cells next to endoderm [86]	-
Annelida	A-D macromeres, foregut and hindgut [68, 201]	hindgut, posterior end [144–148]; 4d-derived bilaterally symmetric lateral cells and early segmental mesoderm [145]	posterior and bilateral bands that can be internalized [144, 150]; teloblasts and ventral nerve cord [150, 212]	posterior end [132, 213]	mesoderm derivatives during larval development [214, 215]	head and and foregut mesoderm [86]	brain, foregut and posterior trunk mesoderm [86]	endo/mesodermal precursors 2Q, 4Q and 4a-c but not 4d [80, 154, 156], endomesoderm and paired lateral bands [155]

taxon	*bra*	*cdx*	*evx*	*wnt1*	*twist*	*foxc*	*foxf*	*gata456*
Nemertea	-	posterior end then subepidermal [120], posterior end and posterior endoderm [121]	posterior end and scattered cells in juvenile [121]	-	larval imaginal discs and broadly later [121]	-	-	larval blind gut and juvenile gut [121]
Brachiopoda	blastopore, foregut [129]	posterior ectoderm and hindgut [129, 216]	hindgut, posterior ectoderm [129]	posterior end of blastopore [208]	mesoderm [87, 129]	apical and pedicle mesoderm, neuroectoderm [87, 129]	anterior mesoderm [87, 129]	endoderm and posterior mesoderm [87, 129]
Phoronida	-	-	-	-	-	-	-	-
Entoprocta	-	-	-	-	-	-	-	-
Bryozoa	posterior ectoderm, larval hindgut	posterior ectoderm, larval hindgut	$2d^{R2}$ posterior ectodermal cell and progeny, 4a, 4c, and 4d mesodermal blastomeres, larval hindgut	posterior end	bilateral mesodermal blastomeres	posterior ectodermal cell, internal sac	anterior larval mesoderm	posterior vegetal blastomeres, larval gut
